# Development of circadian neurovascular function and its implications

**DOI:** 10.3389/fnins.2023.1196606

**Published:** 2023-09-05

**Authors:** Jennifer W. Mitchell, Martha U. Gillette

**Affiliations:** ^1^Department of Cell and Developmental Biology, University of Illinois Urbana-Champaign, Urbana, IL, United States; ^2^Neuroscience Program, University of Illinois Urbana-Champaign, Urbana, IL, United States; ^3^Beckman Institute for Advanced Science and Technology, University of Illinois Urbana-Champaign, Urbana, IL, United States; ^4^Department of Molecular and Integrative Physiology, University of Illinois Urbana-Champaign, Urbana, IL, United States; ^5^Carle-Illinois College of Medicine, University of Illinois Urbana-Champaign, Urbana, IL, United States

**Keywords:** clock, blood–brain interface, neuroendothelial, tight junctions, circadian rhythm disruption

## Abstract

The neurovascular system forms the interface between the tissue of the central nervous system (CNS) and circulating blood. It plays a critical role in regulating movement of ions, small molecules, and cellular regulators into and out of brain tissue and in sustaining brain health. The neurovascular unit (NVU), the cells that form the structural and functional link between cells of the brain and the vasculature, maintains the blood–brain interface (BBI), controls cerebral blood flow, and surveils for injury. The neurovascular system is dynamic; it undergoes tight regulation of biochemical and cellular interactions to balance and support brain function. Development of an intrinsic circadian clock enables the NVU to anticipate rhythmic changes in brain activity and body physiology that occur over the day-night cycle. The development of circadian neurovascular function involves multiple cell types. We address the functional aspects of the circadian clock in the components of the NVU and their effects in regulating neurovascular physiology, including BBI permeability, cerebral blood flow, and inflammation. Disrupting the circadian clock impairs a number of physiological processes associated with the NVU, many of which are correlated with an increased risk of dysfunction and disease. Consequently, understanding the cell biology and physiology of the NVU is critical to diminishing consequences of impaired neurovascular function, including cerebral bleeding and neurodegeneration.

## 1. Introduction

**Figure 1:**
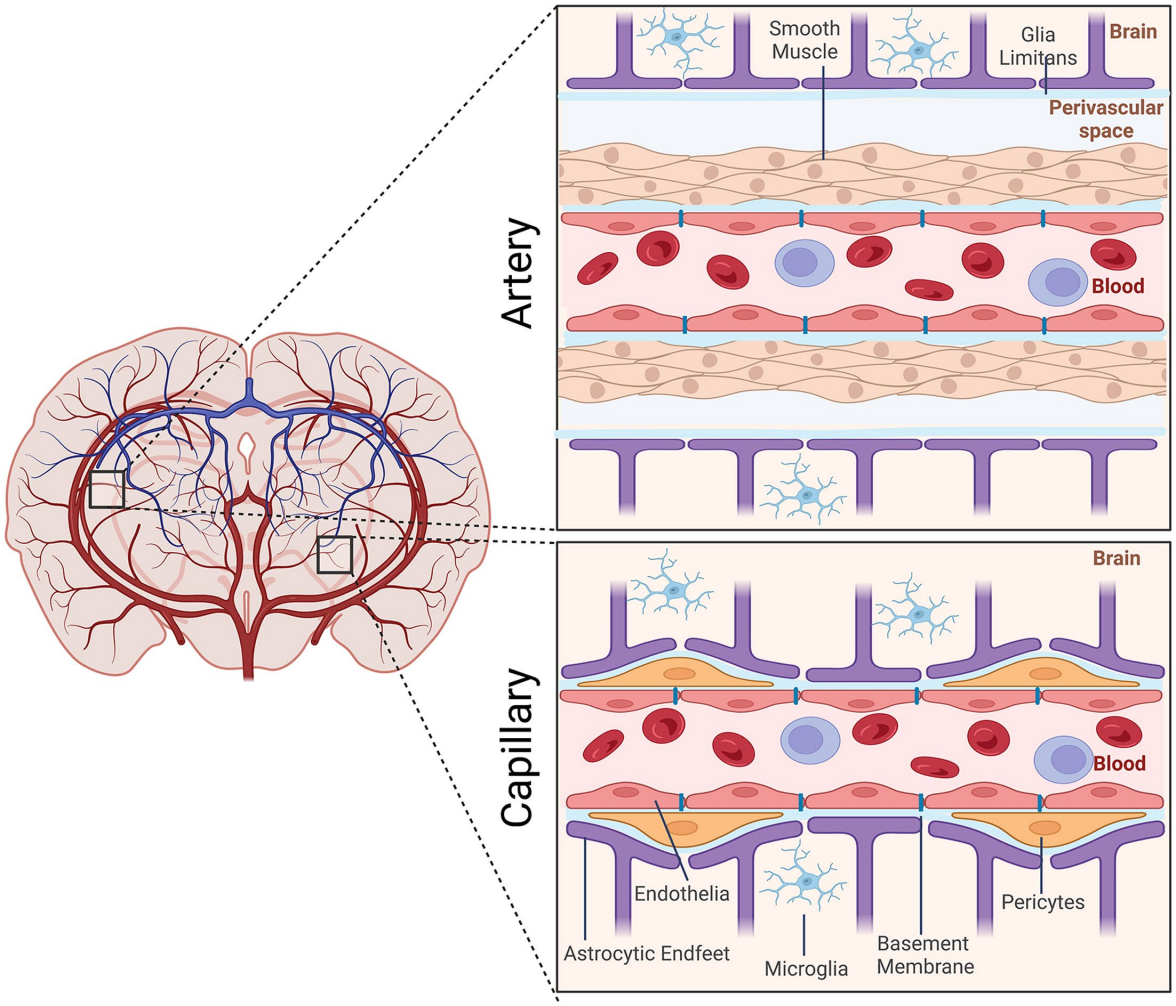
Schematic representation of the cellular elements of the neurovascular unit (NVU). Elements of the NVU include neuroendothelial cells, mural cells (vascular smooth muscle cells, pericytes), microglia, astrocytic endfeet. The cellular composition differs along the vascular tree. At the level of the artery/arteriole (top), the NVU is composed of neuroendothelial cells making up the inner layer of the vessel wall covered by a thin extracellular basement membrane, ringed by vascular smooth muscle cells, and ensheathed by a glial limitans. The perivascular space containing the cerebrospinal fluid is between pia and the glia limitans formed by astrocytic endfeet. At the capillary level (bottom), the NVU is composed of neuroendothelial cells that share a common basement membrane with pericytes. Pericytes stretch their processes along and around capillaries. Pericytes and endothelial cells are covered by astrocyte endfeet. Created by Biorender.com.

The neurovasculature regulates the flow of blood through the arteries, veins, and capillaries within the brain. It is composed of cells of the neurovascular unit (NVU), which include neuroendothelial cells, mural cells (smooth muscle and pericytes), astrocytes, and microglia as well as extracellular matrix components within the basement membrane. Arteries arising from the subarachnoid space vascularize the brain. They form NVUs comprised of smooth muscle cells, endothelial cells, pia mater, the perivascular space, and astrocytic end feet. As these arterial vessels penetrate deeper into the brain, they lose smooth muscle cells and pia mater. Pericytes, which are contractile, assume positions between the endothelial cells and astrocytic endfeet (Figure 1).

The cells of the NVU to undergo dynamic daily and environmental changes, making regulatory adjustments to maintain homeostasis. Interactions of the NVU with neuronal networks are responsible for the regulating cerebral blood flow (CBF), maintaining the integrity of the blood–brain interface (BBI), and immune surveilling-, which recognizes harmful pathogens, responds to injury, recruits resident microglia to clear cellular debris, and releases neuroprotective factors (Kaplan et al., 2020).

### 1.1. Blood–brain interface (BBI)

The cerebrovasculature continuously provides resources supporting the brain’s high metabolic rate, the necessary rapid, on-demand delivery of oxygen and energy supporting neuronal activity, as well as efficient clearance of waste products. The brain’s high metabolic rate requires the continuous supply of nutrients and oxygen by the blood. The blood–brain interface (BBI), a multicellular structure separating the CNS from systemic circulation, regulates this interaction. Tight junctions between endothelial cells of the NVU form a physical barrier, restricting permeability. The surrounding pericytes and astrocytic endfeet are encased in the extracellular matrix-containing basement membrane, enhancing this structural barrier (Abbott et al., 2010; Keaney and Campbell, 2015; Liebner et al., 2018). Vesicular transport across these endothelial cells is very low compared to vascular endothelial cells of other organs, distinguishing them as *neuroendothelium* (Brightman and Reese, 1969). The BBI restricts the exchange of material between the brain and the blood, except for small molecules and gases such as oxygen and carbon dioxide. Larger molecules can cross the BBI only through transporters and endocytic vesicles (pinocytosis). Loss of BBI integrity results not only in reduced oxygen and nutrient flux into the brain, but also diminished clearance of neurotoxic substances. Other components, such as astrocytic endfeet and pericytes, also express some transporters and receptors that contribute to the BBI transport system.

Neuroendothelial cells are the central regulatory components of the BBI. Characterized by few transporters, low level of paracellular diffusion and pinocytotic activity, neuroendothelial cells are joined laterally by tight junctions. Pericytes wrap around the vessels and are embedded in extra cellular matrix, the basement membrane (Abbott et al., 2010). The restrictive properties of the BBI are determined primarily by tight-junction proteins, which form a physical barrier between adjacent neuroendothelial cells (Kniesel and Wolburg, 2000). The BBI restricts entry of cytokines and antibodies, which can impair neurotransmission, into the brain (Abbott et al., 2006), as well as participates in the clearance of cellular metabolites from the brain to the blood (Winkler et al., 2011).

### 1.2. Cerebral blood flow

**Figure 2:**
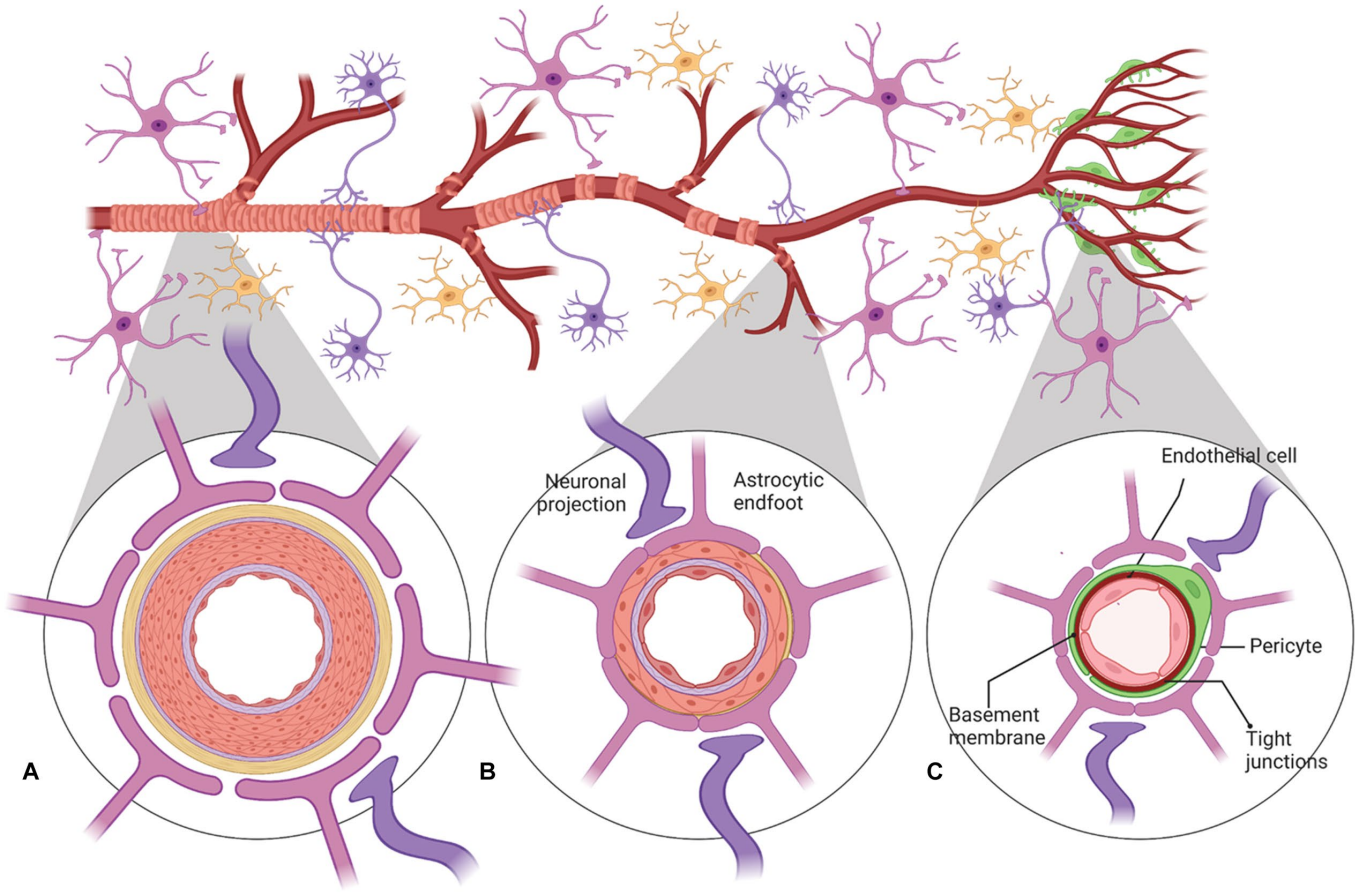
A schematic representation of neurovascular units with cellular elements that regulate cerebral blood flow along the vascular tree. The various types of cells that form the neurovascular unit (NVU) (neurons, astrocytes, mural cells – vascular smooth muscle cells (VSMCs) and pericytes, and neuroendothelium) regulate cerebral blood flow throughout the vascular tree. The cellular composition of the NVU differs along the vascular tree, but the principal cellular components all remain represented, as illustrated here. **(A)** At the level of penetrating arteries, the NVU is composed of neuroendothelial cells, making up the inner layer of the vessel wall, covered by a thin extracellular basement membrane, ringed by one to three layers of VSMCs, and ensheathed by pia. The Virchow-Robin space containing the cerebrospinal fluid is between the pia and glia limitans formed by astrocytic endfeet. Both VSMCs and astrocytes are innervated by local neurons. **(B)** Arterioles differ in that (i) there is only one layer of VSMCs, (ii) astrocyte coverage and innervation of the vessel wall and endothelial inner layer are continuous with penetrating arteries and brain capillaries, above and below the arteriole level, respectively. Precapillary arterioles may also contain transitional pericytes, a cell type between pericyte and VSMCs. **(C)** At the capillary level, the NVU is composed of endothelial cells that share a common basement membrane with pericytes. Pericytes stretch their processes along and around capillaries and make direct, interdigitated or “peg-socket”-like contacts with neuroendothelial cells. Pericytes and neuroendothelial cells are covered by astrocyte endfeet. Both astrocytes and pericytes are innervated by local neurons similar to astrocytes and VSMCs in the upper segments of the vascular tree. Created by Biorender.com.

Cerebral blood flow (CBF) in the human brain is accomplished through a network of interconnected blood vessels over 400-miles long (Zlokovic, 2011; Kisler et al., 2017). Irreversible brain damage can occur within minutes if proper CBF is compromised (Moskowitz et al., 2010). Mural cells, vascular smooth muscle cells (vSMC), and pericytes are involved in regulating cerebral blood flow (Peterson et al., 2011; Hall et al., 2014; Hill et al., 2015; Attwell et al., 2016). Active contraction and relaxation of vSMCs around the larger arterioles controls flow by altering the vascular diameter (Hill et al., 2015). Pericytes are spatially isolated contractile cells in the microvasculature that are responsible for regulating the cerebral blood flow within capillaries (Attwell et al., 2016; Cai et al., 2017; Mughal et al., 2023; Figure 2). The capillary dilation created by pericyte relaxation is regulated by the blood pressure from upstream arterioles. Pericytes alter capillary diameter by actively responding to neuronal activity (Wu et al., 2003; Peppiatt et al., 2006). The dilation response in capillaries is rapid, completing prior to arteriole dilation (Hall et al., 2014).

Regulation of CBF can be grouped into three broad mechanisms: (1) Autoregulation, which maintains stable blood flow despite fluctuations in systemic blood pressure; (2) Vasomotor reactivity, which is in response to modification of the arterial pCO_2_/pH of the brain tissue reporting the need for oxygen; and (3) Neurovascular coupling, which is in response to local changes in neuronal and metabolic activity (Peterson et al., 2011; Silverman and Petersen, 2022). *Autoregulation* is largely controlled by changes in vasoconstriction and vasodilation in the smooth muscle of arterioles (Attwell et al., 2016; Hosford and Gourine, 2019; Hoiland et al., 2020; Duffin et al., 2021). This may be mediated by the release of vasoactive substances, myogenic regulation adapting vascular tone, and neurogenic regulation due to sympathetic innervation of vascular smooth muscle cells (Silverman and Petersen, 2022). *Vasomotor reactivity* reflects changes due to arterial CO_2_ pressure and coupled pH shifts of the brain microenvironment. Small arterioles are extremely sensitive, vasodilating with elevated arterial CO_2_ concentrations (Yoshihara et al., 1995). It has been suggested that this vasomotor response is regulated by the proton concentration in smooth muscle cells of cerebral vessels through the pH sensitivity of carbonic anhydrase activity (Budohoski et al., 2013). *Neurovascular Coupling* responses to elevated neuronal activity and metabolism lead to an increase in local blood flow providing the brain with enhanced blood flow for a given demand (functional hyperemia). Chemical signals secreted from cells of the NVU, which can cause vasodilation or vasoconstriction. The neuroendothelial cells produce several vasoactive factors, including nitric oxide (NO), endothelium-dependent hyperpolarization factor, eicosanoids, and endothelins; their release is regulated by cerebral blood flow (Peterson et al., 2011). Additionally, astrocytic end-feet that directly border the vessels also play key roles in regulating CBF (Peterson et al., 2011). Increased neuronal activity is can initiate the release of synaptic glutamate and thereby activate two different signaling pathways: (1) a signaling pathway that includes the activation of Ca^2+^-dependent enzymes, such as neuronal nitric oxide synthase (nNOS) and cyclooxygenase-2 (COX-2), can produce the vasodilators NO and prostanoids, respectively, and (2) an astrocytic-dependent signaling pathway that includes Ca^2+^-dependent activation of phospholipase A2 and ultimately the production of vasodilatory metabolites including epoxyeicosatrienoic acids (EETs) (Attwell et al., 2010; Stackhouse and Mishra, 2021) (Shi et al., 2008). Additionally, K^+^ and H^+^ are produced during synaptic transmission; elevation of these ions stimulates vasodilation (Paulson and Newman, 1987; Nielsen and Lauritzen, 2001). However, neural activation can also stimulate vasoconstriction as is the case via BK channels from altered astrocytic endfeet calcium signal in brain slices mimicking subarachnoid hemorrhage (Koide et al., 2012; Pappas et al., 2015).

### 1.3. Immune surveillance and repair

Despite barriers that prevent immune cells from traversing into the brain, the CNS is continuously monitoring for damage and agents that would disrupt normal brain function. Resident microglia and immune cells within the bordering meninges are primarily responsible for this surveillance (Ousman and Kubes, 2012). Although the border immune cells are outside the scope of this review, they have been thoroughly discussed (Kwon, 2022; Rustenhoven and Kipnis, 2022). The role of microglia in immune surveillance is facilitated by their close proximity to one another with minimal overlap between processes of neighboring microglia (Raivich, 2005; Ousman and Kubes, 2012). Under physiological conditions, microglia are quiescent (M0), yet they are constantly surveying the local environment and communicating with other cell types via their motile processes. This allows the full extracellular space to be sampled by at least one microglial process every a few hours (Nimmerjahn et al., 2005).

Within the NVU, the microglia are activated by minor alterations to the BBI, responding to various stimuli such as vascular injury, stroke, trauma, bacterial infection as well as diseases states such as Alzheimer’s disease and multiple sclerosis (Dudvarski Stankovic et al., 2016; Orihuela et al., 2016; Zhao et al., 2018). The microglial processes are recruited rapidly and form a dense, continuous, stable aggregate at the site of BBI leakage (Davalos et al., 2012; Jolivel et al., 2015; Lou et al., 2016). Microglia can be activated within minutes of tissue damage. They undergo a spectrum of responses when activated, including polarization and differentiation toward pro-inflammatory and neurotoxicity (M1) or anti-inflammatory, healing (M2) forms (Guo et al., 2022). Microglia driven toward the M1 state can produce inflammatory cytokines and chemokines as well as expressing NADPH oxidase, which produces superoxide and reactive oxygen species (ROS). The M2 state of microglia generate anti-inflammatory cytokines, growth factors, as in insulin-like growth factor 1 (IGF-1) and fibroblastic growth factor (FGF), and neurotropic growth factors, including neurotrophic growth factor (NGF) and brain-derived neurotrophic factor (BDNF). Signals shifting between M1 and M2 polarizations can have profound effects on being either neuroprotective or pathogenetic on brain physiology (Ma et al., 2017; Zhao et al., 2018; Guo et al., 2022).

## 2. Development of the neurovasculature

The developing CNS does not produce vascular progenitor cells; thus, blood vessels must enter into the developing CNS to form the neurovasculature (Bautch and James, 2009). Early studies demonstrate that vascularization of the brain is initiated by vessels outside of the CNS through angiogenic sprouting, namely the peri-neural vascular plexus (Feeney and Watterson, 1946; Strong, 1964). The blood vessels invade and form distinct patterns once they enter the brain (Bautch and James, 2009; Tata et al., 2015). Ultimately, the neurovasculature expands into a vast network and remodels into a vascular tree organized into a hierarchical network of arteries, arterioles, capillary beds, venules, and veins.

Early in embryogenesis, a blood–brain interface is formed to protect neural tissue from variations in blood composition, maintain ionic homeostasis, and exclude toxins. This complex developing structure involves nascent vessels emerging by ingression from endothelial cells. Tight junctions are present between these neuroendothelial cells restricting the passage of low molecular weight molecules very early in development (Ek et al., 2012) The nascent vessels recruit pericytes which are required for barrier formation (Daneman et al., 2010). Notably, this occurs before astrocyte generation (Daneman et al., 2010). Astrocytes extend processes that contact blood vessels after birth, and thus are not required to initially induce the BBI, but likely are involved in maintenance. A number of known signaling molecules between the neural and vascular cells are involved in crosstalk to regulate development of the neurovasculature, all of which allow formation of the intricate architecture through the brain (Tata et al., 2015).

## 3. Specialized neurovascular structures: circumventricular organs and the cerebral portal system

### 3.1. Circumventricular organs

The brain has a number of circumventricular organs (CVOs). CVOs possess characteristically highly permeable, fenestrated capillaries, in contrast to the barrier nature of capillaries of the BBI. As the name implies, these specializations form around (*Lat.*, *circum*) ventricles, including the area postrema (AP), median eminence (ME), neurhypophyis (NP), organum vaculosum of the lamina terminalis (OVLT), pineal gland, and subfornical organ (SFO) (Miyata, 2015). The subcommissural organ (SCO) is often included among the seven CVOs, although it lacks fenestrated capillaries. On the other hand, the choroid plexus, which does have fenestrated capillaries but lacks neurons, may also be grouped with the CVO (Oldfield and McKinley, 2015). Because of the lack of the endothelial barrier, the CVOs permit the direct exchange of chemical information between the brain and circulating blood. Broadly, the CVOs can be separated into two categories, sensory and secretory. The *secretory CVOs* include the NH, ME, and pineal (as well as the SCO&/or choroid plexus). These neurosecretory nuclei release neurohormones and hormone-releasing factors into the blood via the fenestrated capillaries. The *sensory CVOs*, which are often described as “windows of the brain,” detect circulating hormones and ions and initiate responses that maintain homeostasis (Gross et al., 1987) and include the SFO, OVLT and AP.

The presence of these leaky, fenestrated capillaries may allow diffusible timing signals to entrain brain regions outside of the suprachiasmatic nucleus (SCN), the site of the central circadian clock (see section 5). To our knowledge, no prior publication reports movement of signals that traverse from the brain directly into the neurovasculature. However, the dense capillaries within the SCN, specifically, within the shell region, suggest a pathway that transports diffusible signals from the SCN into the blood (Yao et al., 2023). There is evidence that diffusible signals from the SCN can interact locally with the CSF. Taub et al. demonstrated that arginine vasopressin (AVP), an established SCN output timing signal that synchronizes 24-h rhythms of physiology and behavior, was found to contribute to AVP levels in the CSF (Taub et al., 2021). AVP levels exhibit 24-h rhythms in CSF, but not in blood (Schwartz et al., 1983). Previous reports have demonstrated a disparate regulation in neuropeptides between plasma and the CSF (Kagerbauer et al., 2013). However, direct access through CVOs to potential timing signals raises an interesting possibility for regulation of circadian oscillations in the neurovasculature.

### 3.2. Cerebral portal systems

Portal systems carry blood from one capillary bed to another rather than from a capillary bed to a vein. This facilitates the rapid transport of chemical signals from one region to another while maintaining high concentrations of solutes, as the fluid is not transported via the vascular system back through to the heart. The hypophyseal-pituitary portal system is a well-known portal system. Neuropeptides packaged in vesicles within the neuronal cell bodies travel along the axons of the supraoptic and paraventricular neurons that synthesize them to the capillary bed of the median eminence (ME). They are released on demand into the capillary beds of the anterior pituitary (Harris, 1948; Clarke, 2015). Notably, the capillaries and veins of this portal system are leaky due to fenestrations in their vessel walls; thus, the neurohormones pass directly from axon terminals into the blood without encountering a BBI. This portal system allows hypothalamic hormones, such as gonadotropin releasing hormone (GnRH), to stimulate the secretory cells they control.

A new brain portal system has been described that links the SCN and the OVLT (Yao et al., 2021). These portal vessels arise from the rostral SCN and join the capillaries at the base of the OVLT (Yao et al., 2021, 2023). The OVLT participates in osmotic regulation and is involved in the release of AVP (McKinley et al., 2004; Samson and Ferguson, 2015). Many OVLT functions are under circadian control, including anticipatory thirst and osmoregulation (Trudel and Bourque, 2010; Gizowski et al., 2018; Gizowski and Bourque, 2020). The SCN secretes a number of paracrine output signals (Cheng et al., 2002; Kraves and Weitz, 2006; Maywood et al., 2011). This portal system may provide a pathway by which these diffusible circadian-timing signals are maintained at levels that directly influence other brain regions.

## 4. The neurovascular system is dynamic and diurnally rhythmic

Modulation of blood flow, metabolism, and neural activity within the brain is dynamically regulated. The complex multicellular NVU responds accordingly. Beside the changes that occur with aging (Fabiani et al., 2021; Zimmerman et al., 2021), many of these alterations display rhythmic, anticipatory variations that are correlated with specific phases of the 24-h day-night cycle. These circadian rhythms, derived from the Latin *circa* (approximately) and *dies* (day), are driven by endogenous clocks that enable organisms to anticipate and prepare for changes over the day-night cycle. Although not restricted to the neurovasculature, one of the most notable vascular functions that displays circadian rhythmicity is blood pressure (Conroy et al., 2005; Paschos and FitzGerald, 2010). In humans, blood pressure rises before awaking, reaches a peak in midmorning and then decrease as the day progresses (Millar-Craig et al., 1978). Circadian rhythms of regulators of blood pressure, plasma epinephrine, norepinephrine, cortisol, cardiac vagal tone, and heart rate all have been noted (Kalsbeek et al., 2006). Ablation of the hypothalamic SCN disrupts the circadian variation in blood pressure, demonstrating that the central circadian clock is necessary to orchestrate these diverse vascular changes (Janssen et al., 1994; Sano et al., 1995; Witte et al., 1998).

Platelet activation, which mediates blot clot formation, and fibrinolysis, the dissolution of these clots, also displays circadian rhythmicity (Paschos and FitzGerald, 2010; Thosar et al., 2018; Budkowska et al., 2019). With regard to clot formation, platelet aggregation and platelet activation markers show strong time-of-day variation, peaking mid-morning in humans. These include factors promoting blood coagulation, such as platelet factor 4 (PF4), glycoprotein Ib (GPIb), β-thromboglobulin (βTG), P-selectin, and activated integrin αIIbβ3 (also known as GP11b/IIIa) (Tofler et al., 1987; Jafri et al., 1992; Scheer et al., 2011; Thosar et al., 2018; Crnko et al., 2019). Additionally, fibrinogen, the circulating precursor of fibrin, a protein that propagates clotting, demonstrates a 24-h variation in humans (Bremner et al., 2000). In tandem, fibrinolytic activity is significantly reduced in the morning (Andreotti and Kluft, 1991). The concentration of plasminogen activator inhibitor-1 (PAI-1), which inhibits fibrinolysis, peaks in the morning (Andreotti et al., 1988; Kluft et al., 1988; Angleton et al., 1989). This is endogenously driven, independent of the sleep cycle (Scheer and Shea, 2014). Together these findings suggest that mornings, with the increase in platelet activation and decrease in fibrinolysis, constitute an adverse stressor toward negative cardio-and neurovascular events.

Indeed, adverse neurovascular events, including ischemic stroke and hemorrhagic stroke, demonstrate daily fluctuations (Elliott, 1998; Chaturvedi et al., 1999; Manfredini et al., 2005; Butt et al., 2009; Turin et al., 2010; Ripamonti et al., 2017). In intracerebral hemorrhage and subarachnoid hemorrhage detection, a bimodality in the rhythm is present, with the peak in morning and second smaller peak in afternoon/early evening (Butt et al., 2009; Turin et al., 2010). Additionally, differential damage outcome is associated with the time of day of the stroke. In the morning, the increases in paracellular permeability and immune-cell trafficking are associated with more severe stroke phenotype and more adverse outcomes compared to strokes at that occur in the afternoon (Liu et al., 2021). Thus, circadian rhythms may influence stroke vulnerability of the NVU. A more in depth understanding could allow targeted prevention in the development and progression of neurovascular dysfunction and disease.

## 5. The circadian system and the molecular clock

Circadian rhythms are orchestrated by a central pacemaker within the hypothalamus, the SCN. Lesions of the SCN abolish or attenuate daily rhythms in locomotor activity, body temperature, blood pressure, and heart rate in rodents (Stephan and Zucker, 1972; Ralph et al., 1990; Witte et al., 1998), indicating that these are endogenous rhythms rather than driven by 24-h changes in the environment or daily behaviors. SCN rhythms are synchronized to the external environment primarily by light signals via the retinohypothalamic tract (Ding et al., 1994; Gooley et al., 2001). Other salient entrainers, or *zeitgebers*, are food and behavioral activity. The SCN is an autonomous oscillator, maintaining rhythms *in vitro* (Gillette and Reppert, 1987; Yamazaki and Takahashi, 2005). Notably, dissociated SCN cells also remain rhythmic in culture, demonstrating the cell-intrinsic property of rhythmicity (Welsh et al., 2004).

**Figure 3:**
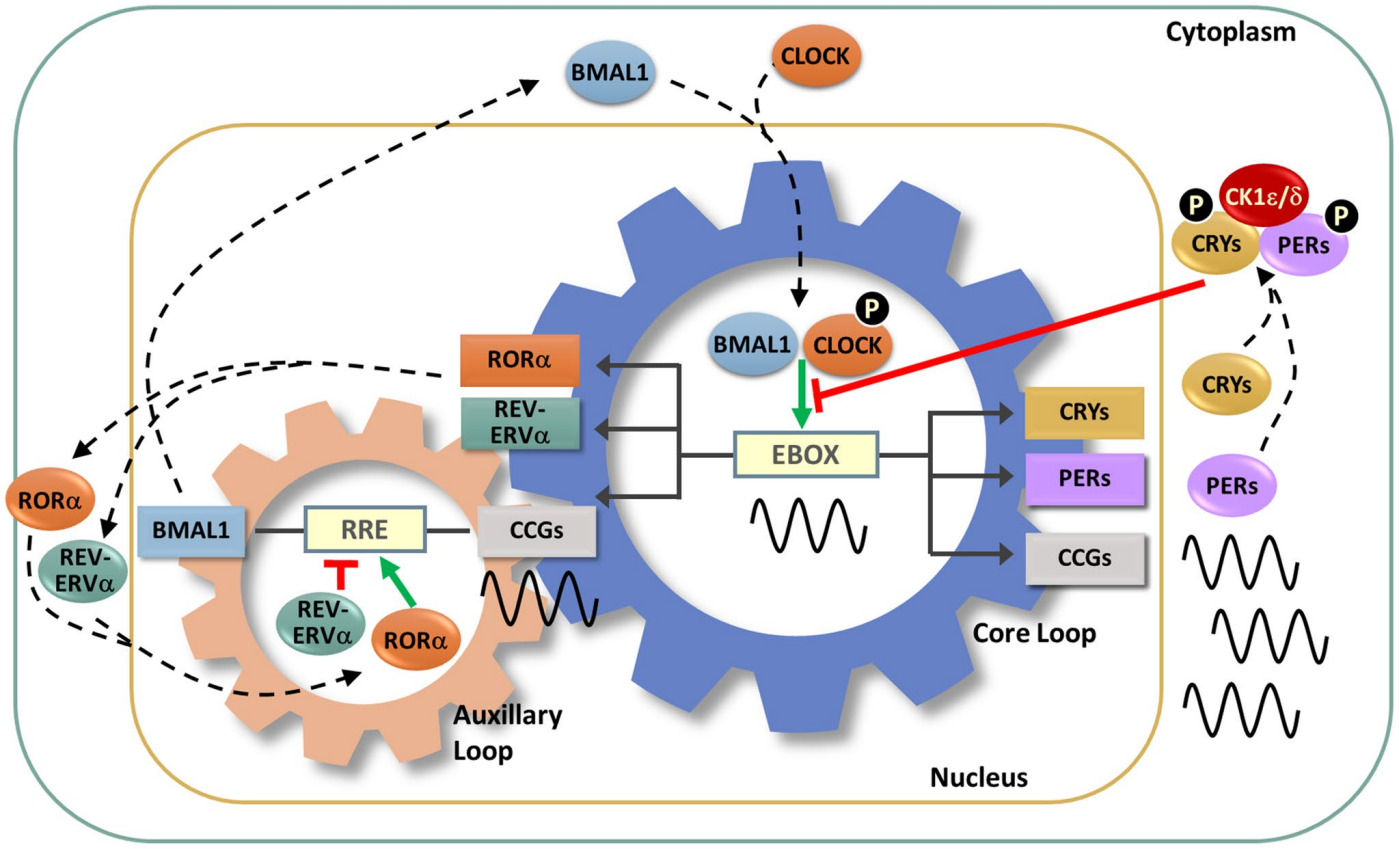
Molecular circadian clockwork is composed of two interlocking transcription/translation feedback loops (TTFLs). The clock proteins, CLOCK and BMAL1, are integral components of the core circadian timekeeping loop. They form a heterodimer, then induce E-box-mediated transcription of negative regulators, *Period* (*Per1,2,3*) and *Cryptochrome* (*Cry1,2*) genes. Accumulated PER and CRY proteins repress E-box-mediated transcription until their levels decrease, allowing the cycle to repeat. CLOCK and BMAL1 also control the transcription of the nuclear receptors RORα and REV-ERBα, which modulate BMAL1 mRNA levels by competitive actions on the RRE element residing in the *Bmal1* promoter. The cycling of clock components collectively determines the temporal patterning and levels of clock-controlled genes (CCGs), thus generating diverse circadian rhythmic outputs. In addition, a number of signaling molecules, including kinases and ubiquitinases, fine-tune these molecular clock loops. Casein kinase Iε (CKIε) and CKIδ form a complex with PERs and CRYs, phosphorylate (P) PERs and then promote proteasome-dependent degradation of these negative regulators. Phosphorylation of CLOCK facilitates its dimerization with BMAL1 and nuclear entry.

At its core, the endogenous circadian clock is a transcriptional-translational negative feedback loop (TTFL). Several interlocking TTFLs act in collaboration with a key negative feedback loop (Figure 3). In this key loop are the positive transcriptional regulators, the proteins BMAL1 and CLOCK. These two positive regulators heterodimerize and bind to *cis*-acting E-box promoter elements to activate the expression of negative regulators, which include the repressors CRYPTOCHROME 1 and 2 (CRY1, CRY2) and PERIOD 1, 2, and 3 (PER1-3). As the cycle progresses, the negative regulators accumulate, form complexes and are transported to the nucleus where they inhibit BMAL/CLOCK-activated transcription, thereby inhibiting their own transcription (Takahashi, 2017; Cox and Takahashi, 2019; Rosensweig and Green, 2020). An auxiliary loop involving REV-ERB and ROR modulates the main TTFL to further controls the expression of CLOCK and BMAL1 (Takahashi, 2017). The regulation and machinery of the molecular clock mechanism has been extensively reviewed (Takahashi, 2017; Cox and Takahashi, 2019; Rosensweig and Green, 2020).

This TTFL generates endogenous circadian rhythms even under constant conditions (absent environmental or behavioral rhythms). These endogenous rhythms are present in nearly all cells and physiological systems, including those of involved with neuronal, metabolic, immune, inflammatory, and vascular function (Thosar et al., 2018, 2019). Collapse of this TTLF leads to internal cellular desynchrony and systemic circadian rhythm disruption. Circadian rhythm disruption results in a higher risk, as well as being an early indicator, for disease (Foster, 2020).

## 6. Circadian influences on cells of the NVU

**Figure 4:**
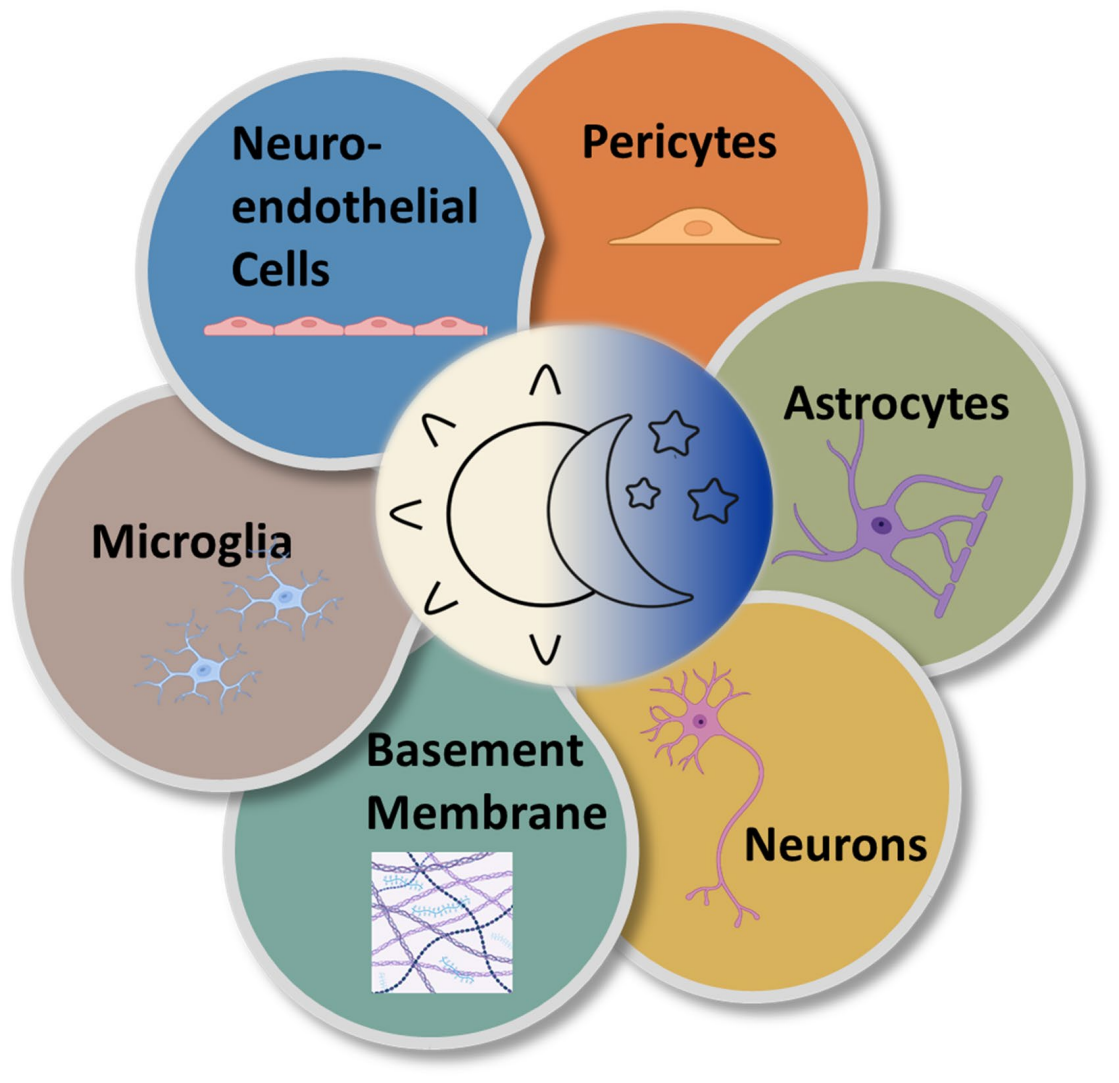
Circadian rhythms are expressed in the various cell types of the NVU. Multiple types of cells comprise the NVU and contribute to neurovascular function. All express near-24-h oscillations in functionality.

Although much of the circadian variation in the neurovascular system is coordinated by the pacemaker function of the SCN, there are also likely local rhythms present within the molecular clocks of the cellular components of the NVU. Each component of the NVU is closely linked. The circadian influences on cells of the NVU are numerous resulting in discrete alterations in these cells across the day night cycle (Figure 4). There is a need to investigate the free-running rhythms in cells of the NVU under constant conditions. Nevertheless, to understand these influences as they currently stand, the various cell types of the NVU will be evaluated individually.

### 6.1. Neuroendothelial cells

The endothelial cells of the BBI are distinct from other endothelial cells. Rather than forming a semi-permeable conduit for the blood, as in the periphery, neuroendothelial cells form a barrier between the brain and the blood, permitting only a few types of molecules to cross. They form a cellular layer with high electrical resistance, tight intercellular junctions, specific transporters, and decreased levels of vesicular transcellular transport compared to peripheral endothelial cells (Betz and Goldstein, 1978; Betz et al., 1980; Daneman, 2012). The activity of efflux transporters is an important regulator of permeability across the BBI. Except in areas of the CVO, there is a general absence of fenestrae in the brain neuroendothelium (Abbott et al., 2010).

Neuroendothelial cells of the luminal membrane of the BBI highly express the ATPase-binding cassette (ABC) efflux transporter, ABCB1, alternatively named permeability glycoprotein (Pgp) and multi-drug resistant protein 1 (MDR1) (Cordon-Cardo et al., 1989; Schinkel et al., 1994). ABCB1 substrate-specific efflux oscillates across the circadian cycle in mouse and human neuroendothelia (Zhang et al., 2021). Although dependent on a functional molecular clock, the ABCB1 transcript family does not exhibit a circadian oscillation nor does the ABC transporter family demonstrate regulation at the transcript level by the circadian clock (Zhang et al., 2021). However, the necessary cofactor in the regulation of ABC transports, intracellular Mg^2+^, demonstrate circadian oscillations that persist in constant conditions in such diverse examples as a human cell line with epithelial morphology, U2OS cells, as well as a unicellular algae, Osteocossus tauri (Feeney et al., 2016). Further, the transient receptor potential cation channel, subfamily M, member 7 (Trpm7), a Mg^2+^ transporter expressed in neuroendothelial cells, exhibits circadian oscillations in its transcript (Zhang et al., 2021). Thus, efflux by transporters, such as ABCB1, may be regulated through its co-factor, via daily fluctuations in intracellular Mg^2+^.

The barrier function of the BBI is regulated by neuroendothelial tight-junction proteins. Tight junctions contain multiple protein complexes, which may include occludins, claudins, zonula occludens (ZO), and junctional adhesion molecules (JAM). Multiple studies have demonstrated circadian regulation of tight proteins at both the RNA and protein levels, although not specifically in the BBI. In retinal pigment epithelium, claudin 2 (cldn2) was found to display circadian rhythms in both protein and RNA expression (Louer et al., 2020). In the inner retinal blood vessels, claudin-5 was found to be highly dynamic and regulated in a circadian manner by the clock protein BMAL1 (Hudson et al., 2019). In intestinal epithelial cells, occludin and claudin-1 mRNA display oscillations across the day-night cycle and their expression is inversely associated with colonic permeability (Oh-oka et al., 2014). This suggests the potential for diurnal or circadian oscillations in the BBI, but this has yet to be determined.

Additionally, neuroendothelial cells can secrete molecules that affect microglia. One such molecule is the proinflammatory cytokine, tumor necrosis factor alpha (TNFα). Although the primary source of TNFα is macrophages and monocytes, TNFα production in neuroendothelial cells has been noted (Imaizumi et al., 2000). TNFα undergoes circadian oscillations in its uptake and can activate interleukin-15 (IL-15). Notably, IL-15 affects the growth of microglia as well as nitric oxide production. Further, IL-15 and its receptor can feedback, exerting anti-inflammatory roles (Pan et al., 2011).

Neuroendothelial cells contribute to the aforementioned circadian fluctuation in the coagulation/fibrinolytic factors. Thrombomodulin, an endothelial membrane protein that exerts anti-coagulation effects through protein C (de Wouwer et al., 2004), displays circadian oscillations at both the level of the RNA and protein in lung and heart (Takeda et al., 2007). Plasminogen activator inhibitor-1 (PAI-1), the principal inhibitor of fibrinolysis *in vivo*, exhibits significant circadian variation with peak circulating levels observed in the morning (Angleton et al., 1989), as well as activation by the core clock proteins, CLOCK and BMAL1 (Schoenhard et al., 2003). Whereas tissue plasminogen antigen (t-PA) which catalyzes the conversion of plasminogen into plasmin and thereby promoter of fibrinolysis, also demonstrated daily variations in levels with increasing concentrations found in the afternoon in humans (Liu et al., 2021).

### 6.2. Astrocytes

The function of astrocytes in the BBI is not fully understood; however, evidence suggests that they induce barrier-like properties in neuroendothelial cells by releasing factors that include TGFβ, glial-derived neurotrophic factor (GDNF), bFGF, and angiopoietin 1 (Abbott et al., 2006), as well as exerting an effect on BBI polarity (Beck et al., 1984). They control neurotransmitter and ion concentrations to maintain homeostatic balance. Astrocytes interface with neuroendothelial cells through the endfeet of specialized processes that line the outer side of cerebral capillaries. They contribute importantly in regulating metabolite levels and brain water content, and they modulate vasodilation. Astrocytes release several molecules that enhance and maintain barrier properties: members of the Hedgehog family, proteins of the renin-angiotensin system, and apolipoprotein (a cholesterol and phospholipid transporter).

Circadian fluctuations in astrocyte structure and function throughout the brain are well documented (McKee et al., 2020; Naseri Kouzehgarani et al., 2022; Rojo et al., 2022; Hastings et al., 2023). Astrocytes contribute to cell–cell coupling in the SCN, undergo daily changes in GFAP distribution and structural complexity, and physical coverage of neurons. Glia respond to daily oscillations in neuronal activity (McKee et al., 2020, Naseri Kouzehgarani et al., 2022, Rojo et al., 2022, Hastings et al., 2023). They may also be involved in thermoregulation, hormonal secretion, and sleep (McKee et al., 2020).

The role of circadian rhythms in astrocyte structure and function in neurovascular function is less understood. However, it is notable that the water channel, aquaporin 4 (AQP4) displays day-night differences in expression levels. AQP4 can control fluid exchange bidirectionally. It is abundant in astrocytes associated with the neurovasculature and is present in high density in astrocytic endfeet bordering blood vessels. Although no difference in the total level of AQP4 has been reported, its polarization to vascular structures is highest during the day in the cortex of rodents suggesting diurnal variation in water exchange across the BBI (Hablitz et al., 2020). Mice deficient in AQP4 display profound reduction in glymphatic clearance (Iliff et al., 2012; Lundgaard et al., 2017), however with regard to BBI integrity, studies lack consensus (Zhou et al., 2008; Saadoun et al., 2009; Haj-Yasein et al., 2011). This topic merits further careful analysis.

### 6.3. Neurons

There is significant heterogeneity in the innervation of the NVU. Broadly, larger, surface extracranial cerebral vessels (such as carotid arteries, vertebral arteries and jugular veins) and intracranial vessels (pial arteries, perforant arteries and pial veins) receive peripheral or extrinsic innervation by cranial autonomic ganglia. Input is derived from sympathetic nerves originating from the superior cervical ganglia, parasympathetic nerves from the sphenopalatine ganglion or otic ganglion, and sensory nerves from the trigeminal ganglion releasing a number of neuropeptides (including substance P, calcitonin gene-related peptide, vasoactive intestinal peptide, pituitary adenylate cyclase-activating peptide, neuropeptide Y, and somatostatin) (Hamel, 2006; Schaeffer and Iadecola, 2021). On the other hand, smaller penetrating arterioles and capillaries receive central or intrinsic innervation via nerve terminals from local interneurons or subcortical pathways, such as the thalamus, locus coeruleus, raphe nucleus and basal forebrain. Communication is mediated via a number of different factors, including neuropeptides, neurotransmitters, prostaglandins, ions, and NO (Giorgi et al., 2020; Schaeffer and Iadecola, 2021).

At the local level, neurons of the NVU directly and indirectly control local cerebral blood flow via neurovascular coupling or function hyperemia in response to neuronal activity, as well as impact vascular networks and BBI formation through release of vasoactive agents (Attwell et al., 2010; Whiteus et al., 2014; McConnell et al., 2017; Kugler et al., 2021). Much of the local neurovascular coupling involved the increased release of glutamate with neuronal activity. Glutamate release can activate neuronal and glial signaling pathways that release vasoactive effectors. Mediators of local neurovascular coupling through inhibitory interneurons have demonstrated the ability to either induce vasodilation or vasoconstriction (Cauli et al., 2004; Rancillac et al., 2006).

Much less is known about the role of circadian neuronal activity in regulating neurovascular function. The numerous neuronal releasates involved in NVU regulation are beyond the scope of this review. We focus on examples with regards to circadian function rather than those involved in acute, inducible alterations to the NVU. Regarding intrinsic innervations that project to cortical microvessels, a number of subcortical pathways are associated with wakefulness and undergo alterations with sleep deprivation. The locus coeruleus expresses 24-h rhythms in the noradrenaline rate-limiting enzyme tyrosine hydroxylase in animals housed under both light:dark (LD) and continuous dark (DD) conditions (Caputo et al., 2023). Additionally, functional MRI in humans has linked cerebral blood flow to diurnal modulation in regions of the default-mode network displaying a decrease in from morning into the afternoon. The default mode network is primarily composed of the medial temporal lobe, the medial prefrontal cortex, and the posterior cingulate cortex and associated with internal thought, active while not engaged in specific tasks (Hodkinson et al., 2014). One of these mediators is nitric active during oxide (NO). NO is generated by a family of three nitric oxide synthase (NOS) isoforms: neuronal NOS (nNOS); endothelial NOS (eNOS), and inducible NOS (iNOS). Although nNOS is abundantly expressed in neurons, it is also expressed in non-neuronal cells (Boulanger et al., 1998; Loesch et al., 1998; Schwarz et al., 1999). It has been argued that the nNOS form participates significantly in regulating microvascular tone (Costa et al., 2016). However, both eNOS and nNOS have been linked to control of vascular tone (Kurihara et al., 1998; Fleming, 2003; Hagioka et al., 2005; Förstermann and Münzel, 2006; Lemmer and Arraj, 2008; Seddon et al., 2008) Multiple genes (*eNOS*, *caveolin1* and *3*) and proteins (tetrahydrobiopterin, phospho-Akt, phospho-eNOS) linked to NO-induced vasodilation display circadian expression, however, their rhythmic regulation in the neurons of the NVU has not been determined (Panda et al., 2002; Rudic et al., 2005; Kunieda et al., 2008; Anea et al., 2009).

Although much research has focused on the effect of neuronal activities on CBF, recent work also has demonstrated an influence of neurons of the NVU on BBI permeability. Elevated expression and function of a major neuroendothelial ABC efflux transporter, P-glycoprotein (pgp), was found to be correlated to decreases in neuronal activity. A diurnal rhythm in pgp substrates efflux was negated in a knockout of the clock gene, Bmal1 (Pulido et al., 2020).

### 6.4. Pericytes

Pericytes are imbedded in the basement membrane in direct contact with neuroendothelial cells, wrapping around the tightly-connected neuroendothelial cells. They are found in the capillary bed but display a marked preference for junctional locations within branching capillaries (Mughal et al., 2023). They have been found to play a regulatory role in brain angiogenesis (the formation of new capillaries from existing ones), neuroendothelial cell-tight junction formation, and differentiation of the blood–brain interface, as well as contribute to structural stability. The CNS vasculature has a significantly higher pericyte coverage compared with peripheral vessels (Winkler et al., 2011; Hall et al., 2014).

Pericytes rhythmically express a number of circadian genes (Mastrullo et al., 2022). A mouse line lacking BMAL1 exhibited a decrease in pericyte coverage within the NVU. This decrease in pericyte coverage increased permeability within the BBI, led to a decrease in platelet-derived growth factor receptor β (PDGFRβ) transcription. The PDGFR is important for BBI integrity (Nakazato et al., 2017b). Exposure to synchronized pericytes in a contact co-culture also synchronizes neuroendothelial circadian rhythms of Bmal1:luciferase, a clock gene reporter (Mastrullo et al., 2022).

### 6.5. Vascular smooth muscle cells

Vascular smooth muscle cells (VSMC) of the NVU are responsible for constriction and dilation of larger arterial and arteriole blood vessels, regulating vascular resistance and blood pressure and stabilizing smaller downstream vessels from the pulsative effect of heartbeat. At this level neurovascular tone is regulated by the relaxation and contraction of VSMC. Hyperpolarization results in the relaxation of the VSMC whereas depolarization results in contraction of the VSMC and constriction of the blood vessels. A number of factors can influence VSMC, including the sympathetic nervous system (discussed in section 6.3) circulating mediators, and endothelial cells (Sandoo et al., 2010; Kisler et al., 2017).

Most work on the influence of the circadian clock in smooth muscle has focused on the peripheral vascular system and not specifically on smooth muscle within neurovascular system. Mesenteric smooth muscle and the aorta exhibit time-of-day variation in contractility in response to vasoconstricting and vasodilating stimuli (Keskil et al., 1996; Witte et al., 2001; Denniff et al., 2014). Molecular clocks have been described in smooth muscle cells (Nonaka et al., 2001) and mesenteric arteries (Denniff et al., 2014). They rhythmically display a number of genes involve in structural integrity in an immortalized vascular smooth muscle cell line, including metalloproteinase 1 and 3 (*timp1*/3), collagen 3a1 (*col3a1*), transgelin 1 (*sm22alpha*), and calponin 1 (*cnn1*) (Chalmers et al., 2008). Deletion of the clock gene, Bmal1, specifically in vascular smooth muscle cells abolishes the circadian variation in pulse pressure and attenuates the amplitude of the daily rhythm in systolic and diastolic blood pressure (Xie et al., 2015). Insights on the molecular circadian clock as well as the effect of a dysfunctional clock specifically on the VSMC within the NVU would be useful, but this has yet to be studied.

### 6.6. Microglia

Microglia are associated with numerous functions within the brain including trophic support, angiogenesis, the regulation of synaptic pruning and neuronal activity (Geloso and D'Ambrosi, 2021; Umpierre and Wu, 2021); however, within the context of the NVU, they are more closely linked to immune surveillance, repairing damage to the BBI as well as regulating vascular tone (Lou et al., 2016; Bisht et al., 2021). Microglia are the resident immune cells in the CNS. In this role, they actively monitor the brain parenchyma via highly motile protrusions and increase phagocytic activity and cytokine production in response to pathogens, neuroinflammation and tissue damage (Madore et al., 2020). Their role in regulating vascular tone involves capillary-associated microglia (CAMS), found in close contact with the capillary wall in the brain in areas not covered by astrocytic endfeet. Loss of CAMS increases blood flow by increasing capillary diameter and results in impaired responses to vasodilation (Bisht et al., 2021).

Microglia have been shown to contain a functional molecular clock and exhibit circadian expression of a number of clock genes, which remain rhythmic in constant conditions, as well as rhythmic expression of a number of microglial markers (Nakazato et al., 2011; Hayashi et al., 2013; Guzman-Ruiz et al., 2023). In addition, Wang et al. demonstrated daily oscillations in the expression of pro-inflammatory cytokines (IL-1β and IL-6) and oxidation-related genes (NADPH oxidase 2, Nox2), as well as molecules involved in nutrient utilization (glucose transporter member 5, Glut5) (Wang et al., 2020). Elevated levels of pro-inflammatory cytokine transcripts during the light (inactive) phase in rodents may indicate a higher innate immune activity when they are sleeping during the day.

Functionally, microglia also exhibit diurnal oscillation in phagocytic activity linked to synaptic pruning during sleep (Choudhury et al., 2020). Furthermore, just as peripheral immune cells display circadian alterations in their response to an immune challenge (Marpegan et al., 2009; Spengler et al., 2012), time-of-day effects within the CNS also have been reported. Microglial process extension are more pronounced at a rodent’s inactive state (day) compared to night (Takayama et al., 2016). Expression of microglial proinflammatory cytokines (IL-1β, TNFα and IL-6) and the inflammatory pathway are elevated with a immune challenge during a rodent’s day (inactive) as compared to night (active) (Fonken et al., 2015; Takayama et al., 2016). It will be important to determine if these day-night differences persist under constant conditions.

Intriguingly, although Cathepsin S (CatS) has not been investigated for a role in altered BBI permeability across the day-night cycle, its demonstrated circadian expression suggests a possible mechanism by which this could occur. CatS, a microglial-specific cysteine protease within the CNS, displays clock-driven expression (Hayashi et al., 2013). CatS-deficient mice exhibited impaired migration across peripheral endothelial basement membranes (Sukhova et al., 2003). It has the ability to degrade ECM molecules at neutral pH (Liuzzo et al., 1999; Vizovišek et al., 2019) and can mediate BBI permeability through proteolytic processing of the junctional adhesion molecule B (JAM-B) (Sevenich et al., 2014).

### 6.7. Basement membrane

The vascular basement membrane, an extracellular matrix (ECM) of structural proteins secreted by cells of the NVU, supports vessel development and maintenance of the BBI. It consists predominately of (1) glycoproteins, such as the integrins laminin, collagen, fibronectin, and vitronectin, (2) enzymes associated with ECM remodeling and processing, and (3) soluble growth factors and cytokines. It is sandwiched between neuroendothelial cells and pericytes on one side and astrocytic endfeet on the other. The ECM proteins are key regulators of cell signaling. The ECM exert its effect through adhesion signaling receptors that sense and report the environment and the surrounding cells (Streuli, 2016).

Although less is known about the role of the ECM in circadian neurovascular function, there is a link of the ECM to clock-gene expression. Changes in the stiffness of the microenvironment of the mammary epithelia can modulate BMAL1-CLOCK activity (Yang et al., 2017). The extracellular environment via integrins transmits signals to the cytoskeleton (Akhtar and Streuli, 2013) and daily oscillations in actin polymerization state have been observed in liver (Gerber et al., 2013) and brain (Gillette, unpublished). The depletion of globular (G) actin toward filamentous (F) actin assemblies release myocardin-related transcription factor (MRTF) from the cytoplasm allowing a rhythmic translocation into the nucleus. MRTF is a serum response factor (SRF) cofactor, and as such leads to 24-h cycles of transcriptional activation by genes targeted though the MRTF-SRF signaling pathway. Thus, a link between the ECM and the circadian molecular clock within the NVU may be through actin dynamics (Streuli and Meng, 2019).

## 7. Impaired neurovascular function with circadian dysregulation

**Figure 5:**
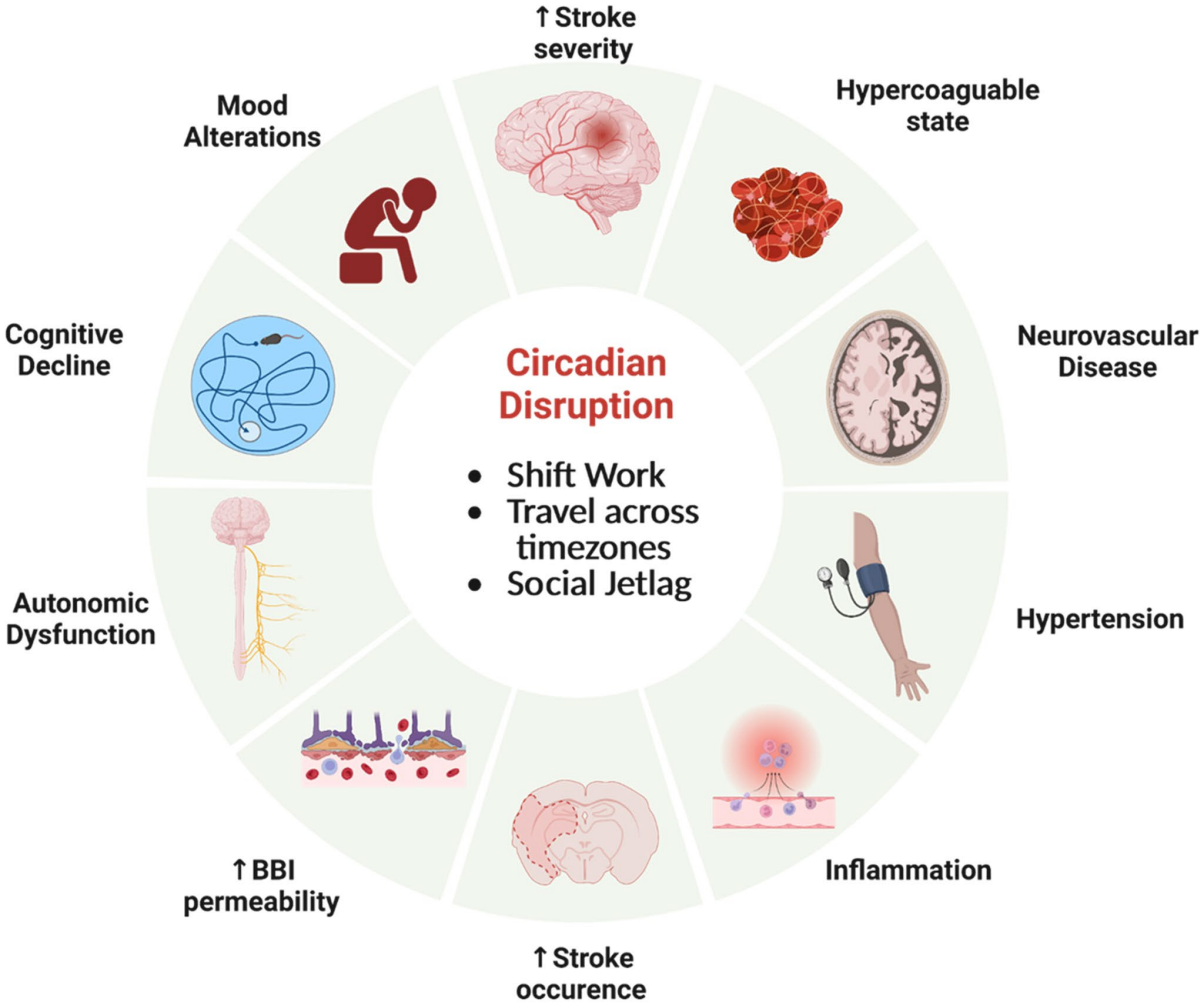
Circadian rhythm disfunction adversely effects physiology. Misalignment of circadian clocks between cells, brain regions, tissue, across the organism and the natural world can increase the risk of significant health consequences involving the neurovasculature and brain. Created by Biorender.com.

Circadian neurovascular function is essential in balancing the influx and efflux between blood and the brain parenchyma; and thereby, modulating the exchange of nutrients and ions as well as protecting neural tissue from access by toxins and pathogens. Circadian rhythm disruption, interference with a stable circadian cycle, can have profound effects on the regulation CNS homeostasis. There has been a rise in human circadian rhythm disruption due to sleep disruption, travel to different time zones, shift work, and social jetlag that has let in an increase in neurological disorders, cancer, metabolic disorders, and mood disorders (Hatori et al., 2017; Lunn et al., 2017; Schurhoff and Toborek, 2023). Alterations of normal circadian rhythms can have profound effects on the neurovasculature, affecting the BBI, cerebral blood flow (CBF), and immune surveillance (Figure 5). These disruptions can contribute to the severity of pathology and response to treatment, most notably with stroke (Thosar et al., 2018; Ramsey et al., 2020; Liu et al., 2021) and traumatic brain injury (Li et al., 2016).

### 7.1. Blood–brain interface (BBI)

The BBI plays an integral role in maintaining tight control of brain chemical composition. Understanding the consequences of alterations to the BBI becomes paramount, considering loss of BBI integrity is present in many neurodegenerative disorders, such as Alzheimer’s disease and Parkinson’s disease (Sweeney et al., 2018). As these diseases involve the accumulation of protein that form fibrils and aggregates, it is important that BBI maintain proper regulation across the interface for removal of waste. Additionally, proteasomal activity follows a circadian rhythm and disruption of these rhythms leads to diminished neuroprotection against protein-plaque formation (Musiek and Holtzman, 2016). Loss of BBI integrity can increase vascular permeability and is associated with cell damage from impaired blood flow and the entrance of toxins into the brain, which can stimulate inflammatory and immune responses. Increases in BBI permeability have been noted with disruption of the molecular clock (Bmal1 knockout) and sleep deprivation (Gómez-González et al., 2013; He et al., 2014; Nakazato et al., 2017b).

With stroke, the blood supply is disrupted, causing essential nutrients not to reach the irreversibly damaged ischemic core, as well as the reversibly injured brain tissue around the ischemic core, the penumbra. Due to an imbalance in ion transport, secondary neuronal damage can occur. Circadian disruptions initiated by weekly phase advances prior to ischemic events exacerbate inflammation and increase in infarct volume (Earnest et al., 2016; Ramsey et al., 2020). It has been suggested that circadian rhythm disruption leads to a propensity for clotting, a prothrombotic state (Liu et al., 2021). A dysfunctional molecular clock in mice, due to either a disruption of *Bmal1* expression or a *Clock* gene mutation, causes a more hypercoagulable state, with alterations in platelet counts and clotting components (Westgate et al., 2008; Hemmeryckx et al., 2011). Considered together, these findings suggest that circadian rhythm dysfunction elevates the risk for greater stroke damage and negative outcome.

### 7.2. Cerebral blood flow

Circadian regulation of blood flow is well documented in the periphery (Wang et al., 1992; Sano et al., 1995; Hermida et al., 2007; Xie et al., 2015; Douma and Gumz, 2018). Blood pressure rises sharply early in the active period and declines to its lowest levels during sleep. However, in a cohort termed “normal dippers,” the mean blood pressure during sleep is 10–20% compared to the daytime-active mean (Hermida et al., 2007). Subjects with a diminished decline in blood pressure (“non-dippers”), have a significantly higher risk for cerebrovascular disease and vascular dementia. Less is known about diurnal alterations in CBF and the consequences of circadian rhythm dysfunction on CBF. Non-invasive measurements of cerebral blood flow velocity (CBFV) in the middle cerebral artery have been used as a surrogate for CBF. Using these methods, daily rhythms in CBFV in human subjects are lower in morning than in afternoon and evening (Sawaya and Ingvar, 1989; Madsen et al., 1991; Droste et al., 1993; Conroy et al., 2005; Kotajima et al., 2005). This phenomenon is often linked with sleep–wake behaviors. CBFV in healthy humans is reduced in non-REM sleep compared to wakefulness (Madsen et al., 1991; Droste et al., 1993; Kotajima et al., 2005). However, Conroy et al. noted time-of-day variations in human CBFV is not altered following 30-h of sustained wakefulness, and therefore not dependent on sleep (Conroy et al., 2005). Similarly in rats, CBF measured a by laser-Doppler flow probe (over 3–4 days) demonstrated diurnal changes independent of peripheral arterial blood pressure or locomotion (Wauschkuhn et al., 2005). These studies were performed under 12:12-h environmental light:dark conditions. This area would benefit from further studies under constant dark conditions to establish the endogenous nature of these oscillations across the circadian cycle.

Regional CBF has been measured by perfusion MRI. CBF in specific regions was altered in workers performing shift work for >2 years. When compared to daytime workers, the cuneus, fusiform/parahippocampal gyri, and cerebellum of shift workers were significantly decreased, while the inferior occipital gyrus was increased (Park et al., 2019). Alterations in circadian rhythms of blood pressure may have significant consequences for cerebral microbleeds. Patients with cerebral microbleeds have higher nocturnal mean systolic blood pressure and lower nocturnal dipping rates (Chen et al., 2022). Patients with cerebral small vessel disease (CSVD) show disturbance of the circadian rhythms in blood pressure (non-dippers and reverse-dippers) as well as cognitive dysfunction (Xu et al., 2023).

### 7.3. Immune surveillance and repair

Disruption of circadian rhythms can have deleterious outcomes for immune surveillance and repair within the brain. Mice exposed to experimental jetlag exhibit increases in inflammatory markers in the blood (Castanon-Cervantes et al., 2010). Circadian rhythm disruption by irregular rest-activity cycles that involve light at night enhances pro-inflammatory cytokine expression following an immune challenge (Fonken et al., 2013). Loss of a functional molecular clock alters microglial immune responses. Pro-inflammatory cytokines were reduced in microglia isolated from Bmal1-deficient mice (Wang et al., 2020). The induction of pro-inflammatory cytokines was attenuated under lipopolysaccharide (LPS) immune challenge of a microglial cell line model with Bmal1 knockdown and in Bmal1-deficient mice (Nakazato et al., 2017a; Wang et al., 2020). Disruption of Rev-erbα caused unprompted microglial activation in the hippocampus increased expression of inflammatory transcripts (*IL-1β/Trem2*) and elevated the inflammatory response to LPS. Together these data suggest that disruption of the circadian clock alters shifts the pro−/anti-inflammatory balance necessary to maintain normal microglial responses to inflammatory challenges.

Circadian rhythm disruptions also can have profound effects on increasing risk and exacerbating disease states. This is likely mediate via changes in immune and inflammatory responses to damage or disease, including to ischemia, traumatic brain injury, and neurodegenerative diseases (i.e., Alzheimer’s disease, Huntington’s disease, and Parkinson’s disease) (Li et al., 2016; Ramsey et al., 2020). Alterations to immune surveillance and repair functions within the NVU have significant effects. For example, in mice subjected to cerebral artery occlusion and reperfusion, then chronic circadian rhythm-disruption group displayed increased infarcts. The interpretation is that this is due to alterations in the ratio of pro-to anti-inflammatory cytokine expression (Ramsey et al., 2020). Although circadian rhythm disruption is known to disrupt peripheral immune responses and to alter circulating proinflammatory cytokines and the complement immune system (Comas et al., 2017; Shivshankar et al., 2020), a defective central immune response also is likely to exacerbate diseases states requiring removal of pathogens and damaged cells in brain.

## 8. Conclusion

The development of the circadian clock in the neurovascular enables anticipation of rhythmic variations that adjust internal states to the 24-h day. In the brain, these changes include oscillations in metabolism, neurohormone secretion, and neurophysiological regulation along with neurovascular components, including cells that form the neurovascular unit and regulate blood flow and platelet aggregation/fibrinolysis. Disruption of the circadian clock has profound effects on mental and physical health. Many disease states are at elevated risk or exacerbated by misalignment of the internal circadian clock (Evans and Davidson, 2013; Foster, 2020; Schurhoff and Toborek, 2023).

Despite significant progress in understanding circadian clocks within the cells of the NVU, knowledge of how the neurovascular units and systems participate in acute responses to cerebrovascular challenges, neurodegenerative diseases, and the aging of the brain is still in its infancy. The roles and risk factors caused by alterations in the circadian system via shift work, travel, and social jet lag are now beginning to be understood. Integrated study of circadian clocks and neurovascular systems has the potential to contribute importantly to understanding cerebral vascular disease, neurodegenerative diseases, and aging, as well as identify isolated key nodes for translational interventions for chronotherapies and other therapeutic benefits.

## Author contributions

JM and MG: drafting and refining the manuscript and critical reading of the manuscript. All authors contributed to the article and approved the submitted version.

## Funding

The authors gratefully acknowledge support in preparation of this manuscript by the National Heart, Lung, and Blood Institute (1R61/R33HL159948) and the Beckman Institute for Advanced Science and Technology.

## Conflict of interest

The authors declare that the research was conducted in the absence of any commercial or financial relationships that could be construed as a potential conflict of interest.

## Publisher’s note

All claims expressed in this article are solely those of the authors and do not necessarily represent those of their affiliated organizations, or those of the publisher, the editors and the reviewers. Any product that may be evaluated in this article, or claim that may be made by its manufacturer, is not guaranteed or endorsed by the publisher.

## References

[ref1] Abbott N. J. Patabendige A. A. Dolman D. E. Yusof S. R. Begley D. J. (2010). Structure and function of the blood-brain barrier. Neurobiol. Dis. 37, 13–25. doi: 10.1016/j.nbd.2009.07.030, PMID: 19664713

[ref2] Abbott N. J. Ronnback L. Hansson E. (2006). Astrocyte-endothelial interactions at the blood-brain barrier. Nat. Rev. Neurosci. 7, 41–53. doi: 10.1038/nrn1824, PMID: 16371949

[ref3] Andreotti F. Davies G. J. Hackett D. R. Khan M. I. De Bart A. C. Aber V. R.. (1988). Major circadian fluctuations in fibrinolytic factors and possible relevance to time of onset of myocardial infarction, sudden cardiac death and stroke. Am. J. Cardiol. 62, 635–637. doi: 10.1016/0002-9149(88)90669-8, PMID: 3137799

[ref4] Andreotti F. Kluft C. (1991). Circadian variation of fibrinolytic activity in blood. Chronobiol. Int. 8, 336–351. 1818784 10.3109/07420529109059170

[ref5] Anea C. B. Zhang M. Stepp D. W. Simkins G. B. Reed G. Fulton D. J.. (2009). Vascular disease in mice with a dysfunctional circadian clock. Circulation 119, 1510–1517. doi: 10.1161/CIRCULATIONAHA.108.827477, PMID: 19273720 PMC2761686

[ref6] Angleton P. Chandler W. L. Schmer G. (1989). Diurnal variation of tissue-type plasminogen activator and its rapid inhibitor (PAI-1). Circulation 79, 101–106. doi: 10.1161/01.CIR.79.1.101, PMID: 2491971

[ref1001] Akhtar N. Streuli C. H. (2013). An integrin–ILK–microtubule network orients cell polarity and lumen formation in glandular epithelium. Nat. cell bio. 15, 17–27. 23263281 10.1038/ncb2646 PMC3701152

[ref7] Attwell D. Buchan A. M. Charpak S. Lauritzen M. Macvicar B. A. Newman E. A. (2010). Glial and neuronal control of brain blood flow. Nature 468, 232–243. doi: 10.1038/nature09613, PMID: 21068832 PMC3206737

[ref8] Attwell D. Mishra A. Hall C. N. O’farrell F. M. Dalkara T. (2016). What is a pericyte? J. Cereb. Blood Flow Metab. 36, 451–455. doi: 10.1177/0271678X15610340, PMID: 26661200 PMC4759679

[ref9] Bautch V. L. James J. M. (2009). Neurovascular development: the beginning of a beautiful friendship. Cell Adhes. Migr. 3, 199–204. doi: 10.4161/cam.3.2.8397 PMC2679887 19363295

[ref10] Beck D. W. Vinters H. V. Hart M. N. Cancilla P. A. (1984). Glial cells influence polarity of the blood-brain barrier. J. Neuropathol. Exp. Neurol. 43, 219–224. doi: 10.1097/00005072-198405000-00001, PMID: 6726282

[ref11] Betz A. L. Firth J. A. Goldstein G. W. (1980). Polarity of the blood-brain barrier: distribution of enzymes between the luminal and antiluminal membranes of brain capillary endothelial cells. Brain Res. 192, 17–28. doi: 10.1016/0006-8993(80)91004-5, PMID: 6103738

[ref12] Betz A. L. Goldstein G. W. (1978). Polarity of the blood-brain barrier: neutral amino acid transport into isolated brain capillaries. Science 202, 225–227. doi: 10.1126/science.211586, PMID: 211586

[ref13] Bisht K. Okojie K. A. Sharma K. Lentferink D. H. Sun Y. Y. Chen H. R.. (2021). Capillary-associated microglia regulate vascular structure and function through PANX1-P2RY12 coupling in mice. Nat. Commun. 12:5289. doi: 10.1038/s41467-021-25590-8 34489419 PMC8421455

[ref14] Boulanger C. M. Heymes C. Benessiano J. Geske R. S. Lévy B. I. Vanhoutte P. M. (1998). Neuronal nitric oxide synthase is expressed in rat vascular smooth muscle cells: activation by angiotensin II in hypertension. Circ. Res. 83, 1271–1278. doi: 10.1161/01.RES.83.12.1271, PMID: 9851944

[ref15] Bremner W. F. Sothern R. B. Kanabrocki E. L. Ryan M. Mccormick J. B. Dawson S.. (2000). Relation between circadian patterns in levels of circulating lipoprotein(a), fibrinogen, platelets, and related lipid variables in men. Am. Heart J. 139, 164–173. doi: 10.1016/S0002-8703(00)90324-7, PMID: 10618578

[ref16] Brightman M. W. Reese T. S. (1969). Junctions between intimately apposed cell membranes in the vertebrate brain. J. Cell Biol. 40, 648–677. doi: 10.1083/jcb.40.3.648, PMID: 5765759 PMC2107650

[ref17] Budkowska M. Lebiecka A. Marcinowska Z. Woźniak J. Jastrzębska M. Dołęgowska B. (2019). The circadian rhythm of selected parameters of the hemostasis system in healthy people. Thromb. Res. 182, 79–88. doi: 10.1016/j.thromres.2019.08.015, PMID: 31473402

[ref18] Budohoski K. P. Czosnyka M. Kirkpatrick P. J. Smielewski P. Steiner L. A. Pickard J. D. (2013). Clinical relevance of cerebral autoregulation following subarachnoid haemorrhage. Nat. Rev. Neurol. 9, 152–163. doi: 10.1038/nrneurol.2013.11, PMID: 23419369

[ref19] Butt M. U. Zakaria M. Hussain H. M. (2009). Circadian pattern of onset of ischaemic and haemorrhagic strokes, and their relation to sleep/wake cycle. J. Pak. Med. Assoc. 59, 129–132. PMID: 19288934

[ref20] Cai W. Liu H. Zhao J. Chen L. Y. Chen J. Lu Z.. (2017). Pericytes in brain injury and repair after ischemic stroke. Transl. Stroke Res. 8, 107–121. doi: 10.1007/s12975-016-0504-4, PMID: 27837475 PMC5350040

[ref21] Caputo R. Poirel V. J. Paiva I. Boutillier A. L. Challet E. Meijer J. H.. (2023). Circadian functioning of locus Cœruleus of the nocturnal rat and diurnal rodent Arvicanthis. Neurosci. Lett. 799:137091. doi: 10.1016/j.neulet.2023.137091, PMID: 36690061

[ref22] Castanon-Cervantes O. Wu M. Ehlen J. C. Paul K. Gamble K. L. Johnson R. L.. (2010). Dysregulation of inflammatory responses by chronic circadian disruption. J. Immunol. 185, 5796–5805. doi: 10.4049/jimmunol.1001026, PMID: 20944004 PMC2974025

[ref23] Cauli B. Tong X. K. Rancillac A. Serluca N. Lambolez B. Rossier J.. (2004). Cortical GABA interneurons in neurovascular coupling: relays for subcortical vasoactive pathways. J. Neurosci. 24, 8940–8949. doi: 10.1523/JNEUROSCI.3065-04.2004, PMID: 15483113 PMC6730057

[ref24] Chalmers J. A. Martino T. A. Tata N. Ralph M. R. Sole M. J. Belsham D. D. (2008). Vascular circadian rhythms in a mouse vascular smooth muscle cell line (Movas-1). Am. J. Phys. Regul. Integr. Comp. Phys. 295, R1529–R1538. doi: 10.1152/ajpregu.90572.2008, PMID: 18768761

[ref25] Chaturvedi S. Adams H. P. Jr. Woolson R. F. (1999). Circadian variation in ischemic stroke subtypes. Stroke 30, 1792–1795. doi: 10.1161/01.STR.30.9.1792, PMID: 10471425

[ref26] Chen Y. K. Liang W. C. Yuan S. L. Ni Z. X. Li W. Liu Y. L.. (2022). Circadian rhythms of blood pressure in hypertensive patients with cerebral microbleeds. Brain Behav. 12:e2530. doi: 10.1002/brb3.2530, PMID: 35234352 PMC9014997

[ref27] Cheng M. Y. Bullock C. M. Li C. Lee A. G. Bermak J. C. Belluzzi J.. (2002). Prokineticin 2 transmits the behavioural circadian rhythm of the suprachiasmatic nucleus. Nature 417, 405–410. doi: 10.1038/417405a, PMID: 12024206

[ref28] Choudhury M. E. Miyanishi K. Takeda H. Islam A. Matsuoka N. Kubo M.. (2020). Phagocytic elimination of synapses by microglia during sleep. Glia 68, 44–59. doi: 10.1002/glia.23698 31429116

[ref29] Clarke I. J. (2015). Hypothalamus as an endocrine organ. Compr. Physiol. 5, 217–253. doi: 10.1002/cphy.c140019 25589270

[ref30] Comas M. Gordon C. J. Oliver B. G. Stow N. W. King G. Sharma P.. (2017). A circadian based inflammatory response–implications for respiratory disease and treatment. Sleep Sci. Pract. 1, 1–19. doi: 10.1186/s41606-017-0019-2

[ref31] Conroy D. A. Spielman A. J. Scott R. Q. (2005). Daily rhythm of cerebral blood flow velocity. J. Circadian Rhythms 3, 1–11. doi: 10.1186/1740-3391-3-3 15760472 PMC555580

[ref32] Cordon-Cardo C. O’brien J. P. Casals D. Rittman-Grauer L. Biedler J. L. Melamed M. R.. (1989). Multidrug-resistance gene (P-glycoprotein) is expressed by endothelial cells at blood-brain barrier sites. Proc. Natl. Acad. Sci. 86, 695–698. doi: 10.1073/pnas.86.2.695, PMID: 2563168 PMC286540

[ref33] Costa E. D. Rezende B. A. Cortes S. F. Lemos V. S. (2016). Neuronal nitric oxide synthase in vascular physiology and diseases. Front. Physiol. 7:206. doi: 10.3389/fphys.2016.00206, PMID: 27313545 PMC4889596

[ref34] Cox K. H. Takahashi J. S. (2019). Circadian clock genes and the transcriptional architecture of the clock mechanism. J. Mol. Endocrinol. 63, R93–R102. doi: 10.1530/JME-19-0153, PMID: 31557726 PMC6872945

[ref35] Crnko S. Du Pré B. C. Sluijter J. P. Van Laake L. W. (2019). Circadian rhythms and the molecular clock in cardiovascular biology and disease. Nat. Rev. Cardiol. 16, 437–447. doi: 10.1038/s41569-019-0167-4, PMID: 30796369

[ref36] Daneman R. (2012). The blood–brain barrier in health and disease. Ann. Neurol. 72, 648–672. doi: 10.1002/ana.23648, PMID: 23280789

[ref37] Daneman R. Zhou L. Kebede A. A. Barres B. A. (2010). Pericytes are required for blood-brain barrier integrity during embryogenesis. Nature 468, 562–566. doi: 10.1038/nature09513, PMID: 20944625 PMC3241506

[ref38] Davalos D. Kyu Ryu J. Merlini M. Baeten K. M. Le Moan N. Petersen M. A.. (2012). Fibrinogen-induced perivascular microglial clustering is required for the development of axonal damage in neuroinflammation. Nat. Commun. 3:1227. doi: 10.1038/ncomms2230 23187627 PMC3514498

[ref39] De Wouwer M. V. Collen D. Conway E. M. (2004). Thrombomodulin-protein C-EPCR system: integrated to regulate coagulation and inflammation. Arterioscler. Thromb. Vasc. Biol. 24, 1374–1383. doi: 10.1161/01.ATV.0000134298.25489.92, PMID: 15178554

[ref40] Denniff M. Turrell H. E. Vanezis A. Rodrigo G. C. (2014). The time-of-day variation in vascular smooth muscle contractility depends on a nitric oxide signalling pathway. J. Mol. Cell. Cardiol. 66, 133–140. doi: 10.1016/j.yjmcc.2013.11.009, PMID: 24262337

[ref41] Ding J. M. Chen D. Weber E. T. Faiman L. E. Rea M. A. Gillette M. U. (1994). Resetting the biological clock: mediation of nocturnal circadian shifts by glutamate and NO. Science 266, 1713–1717. doi: 10.1126/science.7527589, PMID: 7527589

[ref42] Douma L. G. Gumz M. L. (2018). Circadian clock-mediated regulation of blood pressure. Free Radic. Biol. Med. 119, 108–114. doi: 10.1016/j.freeradbiomed.2017.11.024, PMID: 29198725 PMC5910276

[ref43] Droste D. Berger W. Schuler E. Krauss J. (1993). Middle cerebral artery blood flow velocity in healthy persons during wakefulness and sleep: a transcranial Doppler study. Sleep 16, 603–609. PMID: 7904769

[ref44] Dudvarski Stankovic N. Teodorczyk M. Ploen R. Zipp F. Schmidt M. H. (2016). Microglia–blood vessel interactions: a double-edged sword in brain pathologies. Acta Neuropathol. 131, 347–363. doi: 10.1007/s00401-015-1524-y, PMID: 26711460

[ref45] Duffin J. Mikulis D. J. Fisher J. A. (2021). Control of cerebral blood flow by blood gases. Front. Physiol. 12:640075. doi: 10.3389/fphys.2021.640075, PMID: 33679453 PMC7930328

[ref46] Earnest D. J. Neuendorff N. Coffman J. Selvamani A. Sohrabji F. (2016). Sex differences in the impact of shift work schedules on pathological outcomes in an animal model of ischemic stroke. Endocrinology 157, 2836–2843. doi: 10.1210/en.2016-1130, PMID: 27254002 PMC4929545

[ref47] Ek C. J. Dziegielewska K. M. Habgood M. D. Saunders N. R. (2012). Barriers in the developing brain and Neurotoxicology. Neurotoxicology 33, 586–604. doi: 10.1016/j.neuro.2011.12.009 22198708

[ref48] Elliott W. J. (1998). Circadian variation in the timing of stroke onset: a meta-analysis. Stroke 29, 992–996. doi: 10.1161/01.STR.29.5.992, PMID: 9596248

[ref49] Evans J. A. Davidson A. J. (2013). “Chapter ten-health consequences of circadian disruption in humans and animal models” in Progress in molecular biology and translational science. ed. Gillette M. U. (Cambridge, MA: Academic Press) 10.1016/B978-0-12-396971-2.00010-5 23899601

[ref50] Fabiani M. Rypma B. Gratton G. (2021). Aging and cerebrovascular health: structural, functional, cognitive, and methodological implications. Psychophysiology 58:e13842. doi: 10.1111/psyp.13842, PMID: 34021598 PMC8217301

[ref51] Feeney K. A. Hansen L. L. Putker M. Olivares-Yañez C. Day J. Eades L. J.. (2016). Daily magnesium fluxes regulate cellular timekeeping and energy balance. Nature 532, 375–379. doi: 10.1038/nature17407, PMID: 27074515 PMC4886825

[ref52] Feeney J. F. Jr. Watterson R. L. (1946). The development of the vascular pattern within the walls of the central nervous system of the chick embryo. J. Morphol. 78, 231–303. doi: 10.1002/jmor.1050780205, PMID: 21023576

[ref53] Fleming I. (2003). Brain in the brawn: The neuronal nitric oxide synthase as a regulator of myogenic tone. Circ Res 93, 586–588. doi: 10.1161/01.RES.0000095380.06622.D8 14525919

[ref54] Fonken L. K. Frank M. G. Kitt M. M. Barrientos R. M. Watkins L. R. Maier S. F. (2015). Microglia inflammatory responses are controlled by an intrinsic circadian clock. Brain Behav. Immun. 45, 171–179. doi: 10.1016/j.bbi.2014.11.009, PMID: 25433170 PMC4386638

[ref55] Fonken L. K. Weil Z. M. Nelson R. J. (2013). Mice exposed to dim light at night exaggerate inflammatory responses to lipopolysaccharide. Brain Behav. Immun. 34, 159–163. doi: 10.1016/j.bbi.2013.08.011, PMID: 24012645

[ref56] Förstermann U. Münzel T. (2006). Endothelial nitric oxide synthase in vascular disease. Circulation 113, 1708–1714. doi: 10.1161/CIRCULATIONAHA.105.602532, PMID: 16585403

[ref57] Foster R. G. (2020). Sleep, circadian rhythms and health. Interface Focus 10:20190098. doi: 10.1098/rsfs.2019.0098, PMID: 32382406 PMC7202392

[ref58] Geloso M. C. D'ambrosi N. (2021). Microglial pruning: relevance for synaptic dysfunction in multiple sclerosis and related experimental models. Cells 10:686. doi: 10.3390/cells10030686, PMID: 33804596 PMC8003660

[ref59] Gerber A. Esnault C. Aubert G. Treisman R. Pralong F. Schibler U. (2013). Blood-borne circadian signal stimulates daily oscillations in actin dynamics and SRF activity. Cells 152, 492–503. doi: 10.1016/j.cell.2012.12.027, PMID: 23374345

[ref60] Gillette M. U. Reppert S. M. (1987). The hypothalamic suprachiasmatic nuclei: circadian patterns of vasopressin secretion and neuronal activity in vitro. Brain Res. Bull. 19, 135–139. doi: 10.1016/0361-9230(87)90176-6, PMID: 3651837

[ref61] Giorgi F. S. Galgani A. Puglisi-Allegra S. Limanaqi F. Busceti C. L. Fornai F. (2020). Locus Coeruleus and neurovascular unit: from its role in physiology to its potential role in Alzheimer’s disease pathogenesis. J. Neurosci. Res. 98, 2406–2434. doi: 10.1002/jnr.24718, PMID: 32875628

[ref62] Gizowski C. Bourque C. W. (2020). Sodium regulates clock time and output via an excitatory GABAergic pathway. Nature 583, 421–424. doi: 10.1038/s41586-020-2471-x, PMID: 32641825

[ref63] Gizowski C. Zaelzer C. Bourque C. W. (2018). Activation of organum vasculosum neurones and water intake in mice by vasopressin neurones in the suprachiasmatic nucleus. J. Neuroendocrinol. 30:e12577. doi: 10.1111/jne.12577, PMID: 29405459

[ref64] Gómez-González B. Hurtado-Alvarado G. Esqueda-León E. Santana-Miranda R. Rojas-Zamorano J. Velázquez-Moctezuma J. (2013). REM sleep loss and recovery regulates blood-brain barrier function. Curr. Neurovasc. Res. 10, 197–207. doi: 10.2174/15672026113109990002, PMID: 23713739

[ref65] Gooley J. J. Lu J. Chou T. C. Scammell T. E. Saper C. B. (2001). Melanopsin in cells of origin of the retinohypothalamic tract. Nat. Neurosci. 4:1165. doi: 10.1038/nn768, PMID: 11713469

[ref66] Gross P. M. Weindl A. Knigge K. M. (1987). Peering through the windows of the brain. J. Cereb. Blood Flow Metab. 7, 663–672. doi: 10.1038/jcbfm.1987.120, PMID: 2891718

[ref67] Guo S. Wang H. Yin Y. (2022). Microglia polarization from M1 to M2 in neurodegenerative diseases. Front. Aging Neurosci. 14:815347. doi: 10.3389/fnagi.2022.815347 35250543 PMC8888930

[ref68] Guzman-Ruiz M. A. Guerrero-Vargas N. N. Lagunes-Cruz A. Gonzalez-Gonzalez S. Garcia-Aviles J. E. Hurtado-Alvarado G.. (2023). Circadian modulation of microglial physiological processes and immune responses. Glia 71, 155–167. doi: 10.1002/glia.24261, PMID: 35971989 PMC10087862

[ref69] Hablitz L. M. Plá V. Giannetto M. Vinitsky H. S. Stæger F. F. Metcalfe T.. (2020). Circadian control of brain glymphatic and lymphatic fluid flow. Nat. Commun. 11:4411. doi: 10.1038/s41467-020-18115-2 32879313 PMC7468152

[ref70] Hagioka S. Takeda Y. Zhang S. Sato T. Morita K. (2005). Effects of 7-nitroindazole and N-nitro-l-arginine methyl ester on changes in cerebral blood flow and nitric oxide production preceding development of hyperbaric oxygen-induced seizures in rats. Neurosci. Lett. 382, 206–210. doi: 10.1016/j.neulet.2005.01.006, PMID: 15908121

[ref71] Haj-Yasein N. N. Vindedal G. F. Eilert-Olsen M. Gundersen G. A. Skare Ø. Laake P.. (2011). Glial-conditional deletion of aquaporin-4 (Aqp4) reduces blood–brain water uptake and confers barrier function on perivascular astrocyte endfeet. Proc. Natl. Acad. Sci. 108, 17815–17820. doi: 10.1073/pnas.1110655108, PMID: 21990350 PMC3203818

[ref72] Hall C. N. Reynell C. Gesslein B. Hamilton N. B. Mishra A. Sutherland B. A.. (2014). Capillary pericytes regulate cerebral blood flow in health and disease. Nature 508, 55–60. doi: 10.1038/nature13165, PMID: 24670647 PMC3976267

[ref73] Hamel E. (2006). Perivascular nerves and the regulation of cerebrovascular tone. J. Appl. Physiol. 1985, 1059–1064. doi: 10.1152/japplphysiol.00954.2005 16467392

[ref74] Harris G. (1948). Neural control of the pituitary gland. Physiol. Rev. 28, 139–179. doi: 10.1152/physrev.1948.28.2.139, PMID: 18865220

[ref75] Hastings M. H. Brancaccio M. Gonzalez-Aponte M. F. Herzog E. D. (2023). Circadian rhythms and astrocytes: the good, the bad, and the ugly. Annu. Rev. Neurosci. 46, 123–143. doi: 10.1146/annurev-neuro-100322-112249, PMID: 36854316 PMC10381027

[ref76] Hatori M. Gronfier C. Van Gelder R. N. Bernstein P. S. Carreras J. Panda S.. (2017). Global rise of potential health hazards caused by blue light-induced circadian disruption in modern aging societies. NPJ Aging Mech. Dis. 3:9. doi: 10.1038/s41514-017-0010-2 28649427 PMC5473809

[ref77] Hayashi Y. Koyanagi S. Kusunose N. Okada R. Wu Z. Tozaki-Saitoh H.. (2013). The intrinsic microglial molecular clock controls synaptic strength via the circadian expression of cathepsin S. Sci. Rep. 3:2744. doi: 10.1038/srep02744 24067868 PMC3783043

[ref78] He J. Hsuchou H. He Y. Kastin A. J. Wang Y. Pan W. (2014). Sleep restriction impairs blood-brain barrier function. J. Neurosci. 34, 14697–14706. doi: 10.1523/JNEUROSCI.2111-14.2014, PMID: 25355222 PMC4212067

[ref79] Hemmeryckx B. Van Hove C. E. Fransen P. Emmerechts J. Kauskot A. Bult H.. (2011). Progression of the prothrombotic state in aging Bmal1-deficient mice. Arterioscler. Thromb. Vasc. Biol. 31, 2552–2559. doi: 10.1161/ATVBAHA.111.229062, PMID: 21799179

[ref80] Hermida R. C. Ayala D. E. Portaluppi F. (2007). Circadian variation of blood pressure: the basis for the chronotherapy of hypertension. Adv. Drug Deliv. Rev. 59, 904–922. doi: 10.1016/j.addr.2006.08.003, PMID: 17659807

[ref81] Hill R. A. Tong L. Yuan P. Murikinati S. Gupta S. Grutzendler J. (2015). Regional blood flow in the normal and ischemic brain is controlled by arteriolar smooth muscle cell contractility and not by capillary pericytes. Neuron 87, 95–110. doi: 10.1016/j.neuron.2015.06.001, PMID: 26119027 PMC4487786

[ref82] Hodkinson D. J. O’daly O. Zunszain P. A. Pariante C. M. Lazurenko V. Zelaya F. O.. (2014). Circadian and homeostatic modulation of functional connectivity and regional cerebral blood flow in humans under normal entrained conditions. J. Cereb. Blood Flow Metab. 34, 1493–1499. doi: 10.1038/jcbfm.2014.109, PMID: 24938404 PMC4158665

[ref83] Hoiland R. L. Caldwell H. G. Howe C. A. Nowak-Flück D. Stacey B. S. Bailey D. M.. (2020). Nitric oxide is fundamental to neurovascular coupling in humans. J. Physiol. 598, 4927–4939. doi: 10.1113/JP280162, PMID: 32785972

[ref84] Hosford P. S. Gourine A. V. (2019). What is the key mediator of the neurovascular coupling response? Neurosci. Biobehav. Rev. 96, 174–181. doi: 10.1016/j.neubiorev.2018.11.011, PMID: 30481531 PMC6331662

[ref85] Hudson N. Celkova L. Hopkins A. Greene C. Storti F. Ozaki E.. (2019). Dysregulated claudin-5 cycling in the inner retina causes retinal pigment epithelial cell atrophy. JCI insight 4:e130273. doi: 10.1172/jci.insight.130273 31391341 PMC6693834

[ref86] Iliff J. Wang M. Liao Y. Plogg B. Peng W. Gundersen G.. (2012, 2012). A paravascular pathway facilitates CSF flow through the brain parenchyma and the clearance of interstitial solutes, including amyloid β. Sci. Transl. Med. 4:147ra111. doi: 10.1126/scitranslmed.3003748 PMC3551275 22896675

[ref87] Imaizumi T. Itaya H. Fujita K. Kudoh D. Kudoh S. Mori K.. (2000). Expression of tumor necrosis factor-α in cultured human endothelial cells stimulated with lipopolysaccharide or interleukin-1α. Arterioscler. Thromb. Vasc. Biol. 20, 410–415. doi: 10.1161/01.ATV.20.2.410, PMID: 10669637

[ref88] Jafri S. M. Vanrollins M. Ozawa T. Mammen E. F. Goldberg A. D. Goldstein S. (1992). Circadian variation in platelet function in healthy volunteers. Am. J. Cardiol. 69, 951–954. doi: 10.1016/0002-9149(92)90799-5, PMID: 1532287

[ref89] Janssen B. J. Tyssen C. M. Duindam H. Rietveld W. J. (1994). Suprachiasmatic lesions eliminate 24-h blood pressure variability in rats. Physiol. Behav. 55, 307–311. doi: 10.1016/0031-9384(94)90138-4, PMID: 8153170

[ref90] Jolivel V. Bicker F. Binamé F. Ploen R. Keller S. Gollan R.. (2015). Perivascular microglia promote blood vessel disintegration in the ischemic penumbra. Acta Neuropathol. 129, 279–295. doi: 10.1007/s00401-014-1372-1, PMID: 25500713

[ref91] Kagerbauer S. M. Martin J. Schuster T. Blobner M. Kochs E. F. Landgraf R. (2013). Plasma oxytocin and vasopressin do not predict neuropeptide concentrations in human cerebrospinal fluid. J. Neuroendocrinol. 25, 668–673. doi: 10.1111/jne.12038, PMID: 23574490

[ref92] Kalsbeek A. Perreau-Lenz S. Buijs R. M. (2006). A network of (autonomic) clock outputs. Chronobiol. Int. 23, 521–535. doi: 10.1080/07420520600651073 16753939

[ref93] Kaplan L. Chow B. W. Gu C. (2020). Neuronal regulation of the blood–brain barrier and neurovascular coupling. Nat. Rev. Neurosci. 21, 416–432. doi: 10.1038/s41583-020-0322-2, PMID: 32636528 PMC8934575

[ref94] Keaney J. Campbell M. (2015). The dynamic blood-brain barrier. FEBS J. 282, 4067–4079. doi: 10.1111/febs.13412, PMID: 26277326

[ref95] Keskil Z. Gorgun C. Z. Hodoglugil U. Zengil H. (1996). Twenty-four-hour variations in the sensitivity of rat aorta to vasoactive agents. Chronobiol. Int. 13, 465–475. 8974192 10.3109/07420529609020917

[ref96] Kisler K. Nelson A. R. Montagne A. Zlokovic B. V. (2017). Cerebral blood flow regulation and neurovascular dysfunction in Alzheimer disease. Nat. Rev. Neurosci. 18, 419–434. doi: 10.1038/nrn.2017.48, PMID: 28515434 PMC5759779

[ref97] Kluft C. Jie A. Rijken D. Verheijen J. (1988). Daytime fluctuations in blood of tissue-type plasminogen activator (t-PA) and its fast-acting inhibitor (PAI-1). Thromb. Haemost. 59, 329–332. PMID: 3133814

[ref98] Kniesel U. Wolburg H. (2000). Tight junctions of the blood–brain barrier. Cell. Mol. Neurobiol. 20, 57–76. doi: 10.1023/A:1006995910836, PMID: 10690502 PMC11537529

[ref99] Koide M. Bonev A. D. Nelson M. T. Wellman G. C. (2012). Inversion of neurovascular coupling by subarachnoid blood depends on large-conductance Ca2+−activated K+ (BK) channels. Proc. Natl. Acad. Sci. U. S. A. 109, E1387–E1395. doi: 10.1073/pnas.1121359109, PMID: 22547803 PMC3361424

[ref100] Kotajima F. Meadows G. E. Morrell M. J. Corfield D. R. (2005). Cerebral blood flow changes associated with fluctuations in alpha and theta rhythm during sleep onset in humans. J. Physiol. 568, 305–313. doi: 10.1113/jphysiol.2005.092577, PMID: 16002438 PMC1474761

[ref101] Kraves S. Weitz C. J. (2006). A role for cardiotrophin-like cytokine in the circadian control of mammalian locomotor activity. Nat. Neurosci. 9, 212–219. doi: 10.1038/nn1633, PMID: 16429135

[ref102] Kugler E. C. Greenwood J. Macdonald R. B. (2021). The “neuro-glial-vascular” unit: the role of glia in neurovascular unit formation and dysfunction. Front. Cell Develop. Biol. 9:732820. doi: 10.3389/fcell.2021.732820, PMID: 34646826 PMC8502923

[ref103] Kunieda T. Minamino T. Miura K. Katsuno T. Tateno K. Miyauchi H.. (2008). Reduced nitric oxide causes age-associated impairment of circadian rhythmicity. Circ. Res. 102, 607–614. doi: 10.1161/CIRCRESAHA.107.162230, PMID: 18218984

[ref104] Kurihara N. Alfie M. E. Sigmon D. H. Rhaleb N.-E. Shesely E. G. Carretero O. A. (1998). Role of nNOS in blood pressure regulation in eNOS null mutant mice. Hypertension 32, 856–861. doi: 10.1161/01.HYP.32.5.856, PMID: 9822444

[ref105] Kwon D. (2022). Guardians of the brain: how a special immune system protects our grey matter. Nature 606, 22–24. doi: 10.1038/d41586-022-01502-8, PMID: 35650361

[ref106] Lemmer B. Arraj M. (2008). Effect of NO synthase inhibition on cardiovascular circadian rhythms in wild-type and eNOS-knock-out mice. Chronobiol. Int. 25, 501–510. doi: 10.1080/07420520802257695 18622812

[ref107] Li D. Ma S. Guo D. Cheng T. Li H. Tian Y.. (2016). Environmental circadian disruption worsens neurologic impairment and inhibits hippocampal neurogenesis in adult rats after traumatic brain injury. Cell. Mol. Neurobiol. 36, 1045–1055. doi: 10.1007/s10571-015-0295-2, PMID: 26886755 PMC4967018

[ref108] Liebner S. Dijkhuizen R. M. Reiss Y. Plate K. H. Agalliu D. Constantin G. (2018). Functional morphology of the blood-brain barrier in health and disease. Acta Neuropathol. 135, 311–336. doi: 10.1007/s00401-018-1815-1, PMID: 29411111 PMC6781630

[ref109] Liu J. A. Walton J. C. Devries A. C. Nelson R. J. (2021). Disruptions of circadian rhythms and thrombolytic therapy during ischemic stroke intervention. Front. Neurosci. 15:675732. doi: 10.3389/fnins.2021.766648, PMID: 34177452 PMC8222607

[ref110] Liuzzo J. P. Petanceska S. S. Moscatelli D. Devi L. A. (1999). Inflammatory mediators regulate cathepsin S in macrophages and microglia: a role in attenuating heparan sulfate interactions. Mol. Med. 5, 320–333. doi: 10.1007/BF03402068, PMID: 10390548 PMC2230418

[ref111] Loesch A. Milner P. Burnstock G. (1998). Endothelin in perivascular nerves. An electronimmunocytochemical study of rat basilar artery. Neuroreport 9, 3903–3904. doi: 10.1097/00001756-199812010-00025, PMID: 9875726

[ref112] Lou N. Takano T. Pei Y. Xavier A. L. Goldman S. A. Nedergaard M. (2016). Purinergic receptor P2RY12-dependent microglial closure of the injured blood–brain barrier. Proc. Natl. Acad. Sci. 113, 1074–1079. doi: 10.1073/pnas.1520398113, PMID: 26755608 PMC4743790

[ref113] Louer E. M. M. Günzel D. Rosenthal R. Carmone C. Yi G. Stunnenberg H. G.. (2020). Differential day-night expression of tight junction components in murine retinal pigment epithelium. Exp. Eye Res. 193:107985. doi: 10.1016/j.exer.2020.107985, PMID: 32092287

[ref114] Lundgaard I. Lu M. L. Yang E. Peng W. Mestre H. Hitomi E.. (2017). Glymphatic clearance controls state-dependent changes in brain lactate concentration. J. Cereb. Blood Flow Metab. 37, 2112–2124. doi: 10.1177/0271678X16661202, PMID: 27481936 PMC5464705

[ref115] Lunn R. M. Blask D. E. Coogan A. N. Figueiro M. G. Gorman M. R. Hall J. E.. (2017). Health consequences of electric lighting practices in the modern world: a report on the National Toxicology Program's workshop on shift work at night, artificial light at night, and circadian disruption. Sci. Total Environ. 607–608, 1073–1084. doi: 10.1016/j.scitotenv.2017.07.056 PMC5587396 28724246

[ref116] Ma Y. Wang J. Wang Y. Yang G. Y. (2017). The biphasic function of microglia in ischemic stroke. Prog. Neurobiol. 157, 247–272. doi: 10.1016/j.pneurobio.2016.01.005, PMID: 26851161

[ref117] Madore C. Yin Z. Leibowitz J. Butovsky O. (2020). Microglia, lifestyle stress, and neurodegeneration. Immunity 52, 222–240. doi: 10.1016/j.immuni.2019.12.003, PMID: 31924476 PMC7234821

[ref118] Madsen P. L. Holm S. Vorstrup S. Friberg L. Lassen N. A. Wildschiødtz G. (1991). Human regional cerebral blood flow during rapid-eye-movement sleep. J. Cereb. Blood Flow Metab. 11, 502–507. doi: 10.1038/jcbfm.1991.94, PMID: 2016359

[ref119] Manfredini R. Boari B. Smolensky M. H. Salmi R. La Cecilia O. Maria Malagoni A.. (2005). Circadian variation in stroke onset: identical temporal pattern in ischemic and hemorrhagic events. Chronobiol. Int. 22, 417–453. doi: 10.1081/CBI-200062927 16076646

[ref120] Marpegan L. Leone M. J. Katz M. E. Sobrero P. M. Bekinstein T. A. Golombek D. A. (2009). Diurnal variation in endotoxin-induced mortality in mice: correlation with proinflammatory factors. Chronobiol. Int. 26, 1430–1442. doi: 10.3109/07420520903408358, PMID: 19916840

[ref121] Mastrullo V. Van Der Veen D. R. Gupta P. Matos R. S. Johnston J. D. Mcvey J. H.. (2022). Pericytes' circadian clock affects endothelial Cells' synchronization and angiogenesis in a 3D tissue engineered scaffold. Front. Pharmacol. 13:867070. doi: 10.3389/fphar.2022.867070, PMID: 35387328 PMC8977840

[ref122] Maywood E. S. Chesham J. E. O'Brien J. A. Hastings M. H. (2011). A diversity of paracrine signals sustains molecular circadian cycling in suprachiasmatic nucleus circuits. Proc. Natl. Acad. Sci. 108, 14306–14311. doi: 10.1073/pnas.1101767108, PMID: 21788520 PMC3161534

[ref123] Mcconnell H. L. Kersch C. N. Woltjer R. L. Neuwelt E. A. (2017). The translational significance of the neurovascular unit. J. Biol. Chem. 292, 762–770. doi: 10.1074/jbc.R116.760215, PMID: 27920202 PMC5247651

[ref124] Mckee C. A. Lananna B. V. Musiek E. S. (2020). Circadian regulation of astrocyte function: implications for Alzheimer's disease. Cell. Mol. Life Sci. 77, 1049–1058. doi: 10.1007/s00018-019-03314-y, PMID: 31578625 PMC7098845

[ref125] Mckinley M. J. Mathai M. L. Mcallen R. M. Mcclear R. C. Miselis R. R. Pennington G. L.. (2004). Vasopressin secretion: osmotic and hormonal regulation by the lamina terminalis. J. Neuroendocrinol. 16, 340–347. doi: 10.1111/j.0953-8194.2004.01184.x, PMID: 15089972

[ref126] Millar-Craig M. Bishop C. Raftery E. (1978). Circadian variation of blood-pressure. Lancet 311, 795–797. doi: 10.1016/S0140-6736(78)92998-7, PMID: 85815

[ref127] Miyata S. (2015). New aspects in fenestrated capillary and tissue dynamics in the sensory circumventricular organs of adult brains. Front. Neurosci. 9:390. doi: 10.3389/fnins.2015.00390, PMID: 26578857 PMC4621430

[ref128] Moskowitz M. A. Lo E. H. Iadecola C. (2010). The science of stroke: mechanisms in search of treatments. Neuron 67, 181–198. doi: 10.1016/j.neuron.2010.07.002, PMID: 20670828 PMC2957363

[ref129] Mughal A. Nelson M. T. Hill-Eubanks D. (2023). The post-arteriole transitional zone: a specialized capillary region that regulates blood flow within the CNS microvasculature. J. Physiol. 601, 889–901. doi: 10.1113/JP282246, PMID: 36751860 PMC9992301

[ref130] Musiek E. S. Holtzman D. M. (2016). Mechanisms linking circadian clocks, sleep, and neurodegeneration. Science 354, 1004–1008. doi: 10.1126/science.aah4968, PMID: 27885006 PMC5219881

[ref131] Nakazato R. Hotta S. Yamada D. Kou M. Nakamura S. Takahata Y.. (2017a). The intrinsic microglial clock system regulates interleukin-6 expression. Glia 65, 198–208. doi: 10.1002/glia.23087 27726182

[ref132] Nakazato R. Kawabe K. Yamada D. Ikeno S. Mieda M. Shimba S.. (2017b). Disruption of Bmal1 impairs blood-brain barrier integrity via pericyte dysfunction. J. Neurosci. 37, 10052–10062. doi: 10.1523/JNEUROSCI.3639-16.2017 28912161 PMC6596539

[ref133] Nakazato R. Takarada T. Yamamoto T. Hotta S. Hinoi E. Yoneda Y. (2011). Selective upregulation of Per1 mRNA expression by ATP through activation of P2X7 purinergic receptors expressed in microglial cells. J. Pharmacol. Sci. 116, 350–361. doi: 10.1254/jphs.11069FP, PMID: 21747211

[ref134] Naseri Kouzehgarani G. Kandel M. E. Sakakura M. Dupaty J. S. Popescu G. Gillette M. U. (2022). Circadian volume changes in hippocampal glia studied by label-free interferometric imaging. Cells 11:2073. doi: 10.3390/cells11132073 35805157 PMC9265588

[ref135] Nielsen A. N. Lauritzen M. (2001). Coupling and uncoupling of activity-dependent increases of neuronal activity and blood flow in rat somatosensory cortex. J. Physiol. 533, 773–785. doi: 10.1111/j.1469-7793.2001.00773.x, PMID: 11410634 PMC2278665

[ref136] Nimmerjahn A. Kirchhoff F. Helmchen F. (2005). Resting microglial cells are highly dynamic surveillants of brain parenchyma in vivo. Science 308, 1314–1318. doi: 10.1126/science.1110647, PMID: 15831717

[ref137] Nonaka H. Emoto N. Ikeda K. Fukuya H. Rohman M. S. Raharjo S. B.. (2001). Angiotensin II induces circadian gene expression of clock genes in cultured vascular smooth muscle cells. Circulation 104, 1746–1748. doi: 10.1161/hc4001.098048, PMID: 11591607

[ref138] Oh-Oka K. Kono H. Ishimaru K. Miyake K. Kubota T. Ogawa H.. (2014). Expressions of tight junction proteins Occludin and Claudin-1 are under 634 the circadian control in the mouse large intestine: implications in intestinal permeability 635 and susceptibility to colitis. PLoS One 9:636. doi: 10.1371/journal.pone.0098016 PMC4028230 24845399

[ref139] Oldfield B. J. Mckinley M. J. (2015). “Chapter 15- Circumventricular Organs” in The rat nervous system. ed. Paxinos G.. 4th ed (San Diego: Academic Press)

[ref140] Orihuela R. Mcpherson C. A. Harry G. J. (2016). Microglial M1/M2 polarization and metabolic states. Br. J. Pharmacol. 173, 649–665. doi: 10.1111/bph.13139, PMID: 25800044 PMC4742299

[ref141] Ousman S. S. Kubes P. (2012). Immune surveillance in the central nervous system. Nat. Neurosci. 15, 1096–1101. doi: 10.1038/nn.3161, PMID: 22837040 PMC7097282

[ref142] Pan W. Wu X. Kastin A. J. Zhang Y. Hsuchou H. Halberg F.. (2011). Potential protective role of IL15Rα during inflammation. J. Mol. Neurosci. 43, 412–423. doi: 10.1007/s12031-010-9459-1, PMID: 20981579 PMC3521591

[ref143] Panda S. Antoch M. P. Miller B. H. Su A. I. Schook A. B. Straume M.. (2002). Coordinated transcription of key pathways in the mouse by the circadian clock. Cells 109, 307–320. doi: 10.1016/S0092-8674(02)00722-5, PMID: 12015981

[ref144] Pappas A. C. Koide M. Wellman G. C. (2015). Astrocyte Ca2+ signaling drives inversion of neurovascular coupling after subarachnoid hemorrhage. J. Neurosci. 35, 13375–13384. doi: 10.1523/JNEUROSCI.1551-15.2015, PMID: 26424885 PMC4588610

[ref145] Park Y. K. Kim J. H. Choi S. J. Kim S. T. Joo E. Y. (2019). Altered regional cerebral blood flow associated with mood and sleep in shift workers: cerebral perfusion magnetic resonance imaging study. J. Clin. Neurol. 15, 438–447. doi: 10.3988/jcn.2019.15.4.438, PMID: 31591830 PMC6785470

[ref146] Paschos G. K. Fitzgerald G. A. (2010). Circadian clocks and vascular function. Circ. Res. 106, 833–841. doi: 10.1161/CIRCRESAHA.109.211706, PMID: 20299673 PMC2848505

[ref147] Paulson O. B. Newman E. A. (1987). Does the release of potassium from astrocyte endfeet regulate cerebral blood flow? Science 237, 896–898. doi: 10.1126/science.3616619, PMID: 3616619 PMC2505270

[ref148] Peppiatt C. M. Howarth C. Mobbs P. Attwell D. (2006). Bidirectional control of CNS capillary diameter by pericytes. Nature 443, 700–704. doi: 10.1038/nature05193, PMID: 17036005 PMC1761848

[ref149] Peterson E. C. Wang Z. Britz G. (2011). Regulation of cerebral blood flow. J. Vasc. Med. 2011:823525. doi: 10.1155/2011/823525, PMID: 21808738 PMC3144666

[ref150] Pulido R. S. Munji R. N. Chan T. C. Quirk C. R. Weiner G. A. Weger B. D.. (2020). Neuronal activity regulates blood-brain barrier efflux transport through endothelial circadian genes. Neuron 108:e7. doi: 10.1016/j.neuron.2020.09.002 PMC7736535 32979312

[ref151] Raivich G. (2005). Like cops on the beat: the active role of resting microglia. Trends Neurosci. 28, 571–573. doi: 10.1016/j.tins.2005.09.001, PMID: 16165228

[ref152] Ralph M. R. Foster R. G. Davis F. C. Menaker M. (1990). Transplanted suprachiasmatic nucleus determines circadian period. Science 247, 975–978. doi: 10.1126/science.2305266, PMID: 2305266

[ref153] Ramsey A. M. Stowie A. Castanon-Cervantes O. Davidson A. J. (2020). Environmental circadian disruption increases stroke severity and dysregulates immune response. J. Biol. Rhythm. 35, 368–376. doi: 10.1177/0748730420929450, PMID: 32508262 PMC7755461

[ref154] Rancillac A. Rossier J. Guille M. Tong X. K. Geoffroy H. Amatore C.. (2006). Glutamatergic control of microvascular tone by distinct GABA neurons in the cerebellum. J. Neurosci. 26, 6997–7006. doi: 10.1523/JNEUROSCI.5515-05.2006, PMID: 16807329 PMC6673912

[ref155] Ripamonti L. Riva R. Maioli F. Zenesini C. Procaccianti G. (2017). Daily variation in the occurrence of different subtypes of stroke. Stroke Res. Treat 2017:9091250. doi: 10.1155/2017/9091250, PMID: 28717529 PMC5498966

[ref156] Rojo D. Badner A. Gibson E. M. (2022). Circadian control of glial cell homeodynamics. J. Biol. Rhythm. 37, 593–608. doi: 10.1177/07487304221120966, PMID: 36068711 PMC9729367

[ref157] Rosensweig C. Green C. B. (2020). Periodicity, repression, and the molecular architecture of the mammalian circadian clock. Eur. J. Neurosci. 51, 139–165. doi: 10.1111/ejn.14254, PMID: 30402960 PMC6502704

[ref158] Rudic R. D. Mcnamara P. Reilly D. Grosser T. Curtis A.-M. Price T. S.. (2005). Bioinformatic analysis of circadian gene oscillation in mouse aorta. Circulation 112, 2716–2724. doi: 10.1161/CIRCULATIONAHA.105.568626, PMID: 16230482

[ref159] Rustenhoven J. Kipnis J. (2022). Brain borders at the central stage of neuroimmunology. Nature 612, 417–429. doi: 10.1038/s41586-022-05474-7, PMID: 36517712 PMC10205171

[ref160] Saadoun S. Tait M. Reza A. Davies D. C. Bell B. Verkman A.. (2009). AQP4 gene deletion in mice does not alter blood–brain barrier integrity or brain morphology. Neuroscience 161, 764–772. doi: 10.1016/j.neuroscience.2009.03.069, PMID: 19345723

[ref161] Samson W. K. Ferguson A. V. (2015). Exploring the OVLT: insight into a critically important window into the brain. Am. J. Physiol. Regul. Integr. Comp. Physiol. 309, R322–R323. doi: 10.1152/ajpregu.00305.2015, PMID: 26157061

[ref162] Sandoo A. Veldhuijzen Van Zanten J. J. Metsios G. S. Carroll D. Kitas G. D. (2010). The endothelium and its role in regulating vascular tone. Open Cardiovasc. Med. J. 4, 302–312. doi: 10.2174/1874192401004010302, PMID: 21339899 PMC3040999

[ref163] Sano H. Hayashi H. Makino M. Takezawa H. Hirai M. Saito H.. (1995). Effects of suprachiasmatic lesions on circadian rhythms of blood pressure, heart rate and locomotor activity in the rat. Jpn. Circ. J. 59, 565–573. doi: 10.1253/jcj.59.565, PMID: 7474301

[ref164] Sawaya R. Ingvar D. (1989). Cerebral blood flow and metabolism in sleep. Acta Neurol. Scand. 80, 481–491. doi: 10.1111/j.1600-0404.1989.tb03915.x, PMID: 2694726

[ref165] Schaeffer S. Iadecola C. (2021). Revisiting the neurovascular unit. Nat. Neurosci. 24, 1198–1209. doi: 10.1038/s41593-021-00904-7, PMID: 34354283 PMC9462551

[ref166] Scheer F. A. Michelson A. D. Frelinger Iii A. L. Evoniuk H. Kelly E. E. Mccarthy M.. (2011). The human endogenous circadian system causes greatest platelet activation during the biological morning independent of behaviors. PLoS One 6:e24549. doi: 10.1371/journal.pone.0024549, PMID: 21931750 PMC3169622

[ref167] Scheer F. A. J. L. Shea S. A. (2014). Human circadian system causes a morning peak in prothrombotic plasminogen activator inhibitor-1 (PAI-1) independent of the sleep/wake cycle. Blood 123, 590–593. doi: 10.1182/blood-2013-07-517060, PMID: 24200683 PMC3901072

[ref168] Schinkel A. Smit J. Van Tellingen M. Beijnen J. Wagenaar E. Van Deemter L.. (1994). Disruption of the mouse mdr1a P-glycoprotein gene leads to a deficiency in the blood-brain barrier and to increased sensitivity to drugs. Cells 77, 491–502. doi: 10.1016/0092-8674(94)90212-7, PMID: 7910522

[ref169] Schoenhard J. A. Smith L. H. Painter C. A. Eren M. Johnson C. H. Vaughan D. E. (2003). Regulation of the PAI-1 promoter by circadian clock components: differential activation by BMAL1 and BMAL2. J. Mol. Cell. Cardiol. 35, 473–481. doi: 10.1016/S0022-2828(03)00051-8, PMID: 12738229

[ref170] Schurhoff N. Toborek M. (2023). Circadian rhythms in the blood-brain barrier: impact on neurological disorders and stress responses. Mol. Brain 16:5. doi: 10.1186/s13041-023-00997-0 36635730 PMC9835375

[ref171] Schwartz W. J. Coleman R. J. Reppert S. M. (1983). A daily vasopressin rhythm in rat cerebrospinal fluid. Brain Res. 263, 105–112. doi: 10.1016/0006-8993(83)91205-2, PMID: 6839163

[ref172] Schwarz P. M. Kleinert H. FöRstermann U. (1999). Potential functional significance of brain-type and muscle-type nitric oxide synthase I expressed in adventitia and media of rat aorta. Arterioscler. Thromb. Vasc. Biol. 19, 2584–2590. doi: 10.1161/01.ATV.19.11.2584, PMID: 10558999

[ref173] Seddon M. D. Chowienczyk P. J. Brett S. E. Casadei B. Shah A. M. (2008). Neuronal nitric oxide synthase regulates basal microvascular tone in humans in vivo. Circulation 117, 1991–1996. doi: 10.1161/CIRCULATIONAHA.107.744540, PMID: 18391107

[ref174] Sevenich L. Bowman R. L. Mason S. D. Quail D. F. Rapaport F. Elie B. T.. (2014). Analysis of tumour-and stroma-supplied proteolytic networks reveals a brain-metastasis-promoting role for cathepsin S. Nat. Cell Biol. 16, 876–888. doi: 10.1038/ncb3011, PMID: 25086747 PMC4249762

[ref175] Shi Y. Liu X. Gebremedhin D. Falck J. R. Harder D. R. Koehler R. C. (2008). Interaction of mechanisms involving epoxyeicosatrienoic acids, adenosine receptors, and metabotropic glutamate receptors in neurovascular coupling in rat whisker barrel cortex. J. Cereb. Blood Flow Metab. 28, 111–125. doi: 10.1038/sj.jcbfm.9600511, PMID: 17519974 PMC2204069

[ref176] Shivshankar P. Fekry B. Eckel-Mahan K. Wetsel R. A. (2020). Circadian clock and complement immune system—complementary control of physiology and pathology? Front. Cell. Infect. Microbiol. 10:418. 32923410 10.3389/fcimb.2020.00418 PMC7456827

[ref177] Silverman A. Petersen N. H. (2022). Physiology, cerebral autoregulation, StatPearls Treasure Island, FL. 31985976

[ref178] Spengler M. L. Kuropatwinski K. K. Comas M. Gasparian A. V. Fedtsova N. Gleiberman A. S.. (2012). Core circadian protein CLOCK is a positive regulator of NF-κB–mediated transcription. Proc. Natl. Acad. Sci. 109, E2457–E2465. doi: 10.1073/pnas.1206274109, PMID: 22895791 PMC3443185

[ref179] Stackhouse T. L. Mishra A. (2021). Neurovascular coupling in development and disease: focus on astrocytes. Front. Cell Devel. Biol. 9:702832. doi: 10.3389/fcell.2021.702832, PMID: 34327206 PMC8313501

[ref180] Stephan F. K. Zucker I. (1972). Circadian rhythms in drinking behavior and locomotor activity of rats are eliminated by hypothalamic lesions. Proc. Natl. Acad. Sci. 69, 1583–1586. doi: 10.1073/pnas.69.6.1583, PMID: 4556464 PMC426753

[ref181] Streuli C. H. (2016). Integrins as architects of cell behavior. Mol. Biol. Cell 27, 2885–2888. doi: 10.1091/mbc.E15-06-0369, PMID: 27687254 PMC5042575

[ref182] Streuli C. H. Meng Q. J. (2019). Influence of the extracellular matrix on cell-intrinsic circadian clocks. J. Cell Sci. 132:jcs207498. doi: 10.1242/jcs.207498, PMID: 30709969

[ref183] Strong L. H. (1964). The early embryonic pattern of internal vascularization of the mammalian cerebral cortex. J. Comp. Neurol. 123, 121–138. doi: 10.1002/cne.901230111, PMID: 14199263

[ref184] Sukhova G. K. Zhang Y. Pan J. H. Wada Y. Yamamoto T. Naito M.. (2003). Deficiency of cathepsin S reduces atherosclerosis in LDL receptor-deficient mice. J. Clin. Invest. 111, 897–906. doi: 10.1172/JCI200314915, PMID: 12639996 PMC153760

[ref185] Sweeney M. D. Sagare A. P. Zlokovic B. V. (2018). Blood–brain barrier breakdown in Alzheimer disease and other neurodegenerative disorders. Nat. Rev. Neurol. 14, 133–150. doi: 10.1038/nrneurol.2017.188, PMID: 29377008 PMC5829048

[ref186] Takahashi J. S. (2017). Transcriptional architecture of the mammalian circadian clock. Nat. Rev. Genet. 18, 164–179. doi: 10.1038/nrg.2016.150, PMID: 27990019 PMC5501165

[ref187] Takayama F. Hayashi Y. Wu Z. Liu Y. Nakanishi H. (2016). Diurnal dynamic behavior of microglia in response to infected bacteria through the UDP-P2Y6 receptor system. Sci. Rep. 6, 1–10. doi: 10.1038/srep30006 27445174 PMC4956748

[ref188] Takeda N. Maemura K. Horie S. Oishi K. Imai Y. Harada T.. (2007). Thrombomodulin is a clock-controlled gene in vascular endothelial cells. J. Biol. Chem. 282, 32561–32567. doi: 10.1074/jbc.M705692200, PMID: 17848551

[ref189] Tata M. Ruhrberg C. Fantin A. (2015). Vascularisation of the central nervous system. Mech. Dev. 138, 26–36. doi: 10.1016/j.mod.2015.07.001 26222953 PMC4678116

[ref190] Taub A. Carbajal Y. Rimu K. Holt R. Yao Y. Hernandez A. L.. (2021). Arginine vasopressin-containing neurons of the suprachiasmatic nucleus project to CSF. eNeuro 8:ENEURO.0363-20.2021. doi: 10.1523/ENEURO.0363-20.2021, PMID: 33472866 PMC8174031

[ref191] Thosar S. S. Berman A. M. Herzig M. X. Mchill A. W. Bowles N. P. Swanson C. M.. (2019). Circadian rhythm of vascular function in midlife adults. Arterioscler. Thromb. Vasc. Biol. 39, 1203–1211. doi: 10.1161/ATVBAHA.119.312682, PMID: 31070470 PMC6531330

[ref192] Thosar S. S. Butler M. P. Shea S. A. (2018). Role of the circadian system in cardiovascular disease. J. Clin. Invest. 128, 2157–2167. doi: 10.1172/JCI80590 29856365 PMC5983320

[ref193] Tofler G. H. Brezinski D. Schafer A. I. Czeisler C. A. Rutherford J. D. Willich S. N.. (1987). Concurrent morning increase in platelet aggregability and the risk of myocardial infarction and sudden cardiac death. N. Engl. J. Med. 316, 1514–1518. doi: 10.1056/NEJM198706113162405, PMID: 3587281

[ref194] Trudel E. Bourque C. W. (2010). Central clock excites vasopressin neurons by waking osmosensory afferents during late sleep. Nat. Neurosci. 13, 467–474. doi: 10.1038/nn.2503, PMID: 20190744

[ref195] Turin T. C. Kita Y. Rumana N. Takashima N. Ichikawa M. Sugihara H.. (2010). Diurnal variation in onset of hemorrhagic stroke is independent of risk factor status: Takashima stroke registry. Neuroepidemiology 34, 25–33. doi: 10.1159/000255463, PMID: 19893326

[ref196] Umpierre A. D. Wu L. J. (2021). How microglia sense and regulate neuronal activity. Glia 69, 1637–1653. doi: 10.1002/glia.23961, PMID: 33369790 PMC8113084

[ref197] Vizovišek M. Fonović M. Turk B. (2019). Cysteine cathepsins in extracellular matrix remodeling: extracellular matrix degradation and beyond. Matrix Biol. 75, 141–159. doi: 10.1016/j.matbio.2018.01.024 29409929

[ref198] Wang Z. Wang L. Zhang L. Liu Q. Xue Z. Cornélissen G.. (1992). Circadian relations among cardiovascular variables of young adults. Chronobiologia 19, 111–120. PMID: 1478112

[ref199] Wang X. L. Wolff S. E. C. Korpel N. Milanova I. Sandu C. Rensen P. C. N.. (2020). Deficiency of the circadian clock gene Bmal1 reduces microglial Immunometabolism. Front. Immunol. 11:586399. doi: 10.3389/fimmu.2020.586399, PMID: 33363534 PMC7753637

[ref200] Wauschkuhn C. A. Witte K. Gorbey S. Lemmer B. Schilling L. (2005). Circadian periodicity of cerebral blood flow revealed by laser-Doppler flowmetry in awake rats: relation to blood pressure and activity. Am. J. Phys. Heart Circ. Phys. 289, H1662–H1668. doi: 10.1152/ajpheart.01242.2004, PMID: 15894567

[ref201] Welsh D. K. Yoo S. H. Liu A. C. Takahashi J. S. Kay S. A. (2004). Bioluminescence imaging of individual fibroblasts reveals persistent, independently phased circadian rhythms of clock gene expression. Curr. Biol. 14, 2289–2295. doi: 10.1016/j.cub.2004.11.057, PMID: 15620658 PMC3777438

[ref202] Westgate E. J. Cheng Y. Reilly D. F. Price T. S. Walisser J. A. Bradfield C. A.. (2008). Genetic components of the circadian clock regulate thrombogenesis in vivo. Circulation 117, 2087–2095. doi: 10.1161/CIRCULATIONAHA.107.739227, PMID: 18413500

[ref203] Whiteus C. Freitas C. Grutzendler J. (2014). Perturbed neural activity disrupts cerebral angiogenesis during a postnatal critical period. Nature 505, 407–411. doi: 10.1038/nature12821, PMID: 24305053 PMC3947100

[ref204] Winkler E. A. Bell R. D. Zlokovic B. V. (2011). Central nervous system pericytes in health and disease. Nat. Neurosci. 14, 1398–1405. doi: 10.1038/nn.2946, PMID: 22030551 PMC4020628

[ref205] Witte K. Hasenberg T. Rueff T. Hauptfleisch S. Schilling L. Lemmer B. (2001). Day-night variation in the in vitro contractility of aorta and mesenteric and renal arteries in transgenic hypertensive rats. Chronobiol. Int. 18, 665–681. 11587089 10.1081/cbi-100106080

[ref206] Witte K. Schnecko A. Buijs R. M. Van Der Vliet J. Scalbert E. Delagrange P.. (1998). Effects of Scn lesions on orcadian blood pressure rhythm in normotensive and transgenic hypertensive rats. Chronobiol. Int. 15, 135–145. doi: 10.1081/cbi-100106080 9562918

[ref207] Wu D. M. Kawamura H. Sakagami K. Kobayashi M. Puro D. G. (2003). Cholinergic regulation of pericyte-containing retinal microvessels. Am. J. Phys. Heart Circ. Phys. 284, H2083–H2090. doi: 10.1152/ajpheart.01007.2002, PMID: 12560212

[ref208] Xie Z. Su W. Liu S. Zhao G. Esser K. Schroder E. A.. (2015). Smooth-muscle BMAL1 participates in blood pressure circadian rhythm regulation. J. Clin. Invest. 125, 324–336. doi: 10.1172/JCI76881, PMID: 25485682 PMC4382248

[ref209] Xu Y. Gong C. Liao J. Ge Z. Tan Y. Jiang Y.. (2023). Absence of fluctuation and inverted circadian rhythm of blood pressure increase the risk of cognitive dysfunction in cerebral small vessel disease patients. BMC Neurol. 23:73. doi: 10.1186/s12883-023-03107-8 36793019 PMC9930256

[ref210] Yamazaki S. Takahashi J. S. (2005). Real-time luminescence reporting of circadian gene expression in mammals. Methods Enzymol. 393, 288–301. doi: 10.1016/S0076-6879(05)93012-7 15817295 PMC3793321

[ref211] Yang N. Williams J. Pekovic-Vaughan V. Wang P. Olabi S. Mcconnell J.. (2017). Cellular mechano-environment regulates the mammary circadian clock. Nat. Commun. 8:14287. doi: 10.1038/ncomms14287 28134247 PMC5290282

[ref212] Yao Y. Green I. K. Taub A. B. Tazebay R. Lesauter J. Silver R. (2023). Vasculature of the suprachiasmatic nucleus: pathways for diffusible output signals. J. Biol. Rhythms. 8:7487304231189537. doi: 10.1177/07487304231189537, PMID: 37553858 PMC10652420

[ref213] Yao Y. Taub A. B. N. Lesauter J. Silver R. (2021). Identification of the suprachiasmatic nucleus venous portal system in the mammalian brain. Nat. Commun. 12:5643. doi: 10.1038/s41467-021-25793-z 34561434 PMC8463669

[ref214] Yoshihara M. Bandoh K. Marmarou A. (1995). Cerebrovascular carbon dioxide reactivity assessed by intracranial pressure dynamics in severely head injured patients. J. Neurosurg. 82, 386–393. doi: 10.3171/jns.1995.82.3.0386, PMID: 7861215

[ref215] Zhang S. L. Lahens N. F. Yue Z. Arnold D. M. Pakstis P. P. Schwarz J. E.. (2021). A circadian clock regulates efflux by the blood-brain barrier in mice and human cells. Nat. Commun. 12:617. doi: 10.1038/s41467-020-20795-9 33504784 PMC7841146

[ref216] Zhao X. Eyo U. B. Murugan M. Wu L. J. (2018). Microglial interactions with the neurovascular system in physiology and pathology. Dev. Neurobiol. 78, 604–617. doi: 10.1002/dneu.22576, PMID: 29318762 PMC5980686

[ref217] Zhou J. Kong H. Hua X. Xiao M. Ding J. Hu G. (2008). Altered blood–brain barrier integrity in adult aquaporin-4 knockout mice. Neuroreport 19, 1–5. doi: 10.1097/WNR.0b013e3282f2b4eb, PMID: 18281883

[ref218] Zimmerman B. Rypma B. Gratton G. Fabiani M. (2021). Age-related changes in cerebrovascular health and their effects on neural function and cognition: a comprehensive review. Psychophysiology 58:e13796. doi: 10.1111/psyp.13796, PMID: 33728712 PMC8244108

[ref219] Zlokovic B. V. (2011). Neurovascular pathways to neurodegeneration in Alzheimer's disease and other disorders. Nat. Rev. Neurosci. 12, 723–738. doi: 10.1038/nrn3114, PMID: 22048062 PMC4036520

